# STARD7 maintains intestinal epithelial mitochondria architecture, barrier integrity, and protection from colitis

**DOI:** 10.1172/jci.insight.172978

**Published:** 2024-11-22

**Authors:** Jazib Uddin, Ankit Sharma, David Wu, Sunil Tomar, Varsha Ganesan, Paula E. Reichel, Lakshmi Narasimha Rao Thota, Rodolfo I. Cabrera-Silva, Sahiti Marella, Gila Idelman, Hock L. Tay, Arturo Raya-Sandino, Mack B. Reynolds, Srikanth Elesela, Yael Haberman, Lee A. Denson, Charles A. Parkos, Mary X.D. O’Riordan, Nicholas W. Lukacs, David N. O’Dwyer, Senad Divanovic, Asma Nusrat, Timothy E. Weaver, Simon P. Hogan

**Affiliations:** 1 Division of Experimental Pathology, Department of Pathology, and; 2 Graduate Program in Immunology, University of Michigan, Ann Arbor, Michigan, USA.; 3 Division of Allergy and Immunology, Cincinnati Children’s Hospital Medical Center, University of Cincinnati College of Medicine, Cincinnati, Ohio, USA.; 4 Department of Microbiology and Immunology, University of Michigan Medical School, Ann Arbor, Michigan, USA.; 5 Sheba Medical Center, Tel-Hashomer, and; 6 Faculty of Medicine, Tel Aviv University, Tel Aviv, Israel.; 7 Division of Pediatric Gastroenterology, Hepatology and Nutrition, Department of Pediatrics, Cincinnati Children’s Hospital Medical Center, University of Cincinnati College of Medicine, Cincinnati, Ohio, USA.; 8 Mary H. Weiser Food Allergy Center, Michigan Medicine, University of Michigan, Ann Arbor, Michigan, USA.; 9 Department of Internal Medicine, University of Michigan Medical School, Ann Arbor, Michigan, USA.; 10 Division of Immunobiology, Department of Pediatrics, Cincinnati Children’s Hospital Medical Center, University of Cincinnati College of Medicine, Cincinnati, Ohio, USA.; 11 Center for Inflammation and Tolerance and; 12 Divisions of Neonatology, Perinatal Biology, and Pulmonary Biology, Perinatal Institute, Cincinnati Children’s Hospital Medical Center, Cincinnati, Ohio, USA.

**Keywords:** Inflammation, Inflammatory bowel disease, Mitochondria

## Abstract

Susceptibility to inflammatory bowel diseases (IBDs), Crohn’s disease (CD), and ulcerative colitis (UC) is linked with loss of intestinal epithelial barrier integrity and mitochondria dysfunction. Steroidogenic acute regulatory (StAR) protein-related lipid transfer (START) domain-containing protein 7 (STARD7) is a phosphatidylcholine-specific (PC-specific) lipid transfer protein that transports PC from the ER to the mitochondria, facilitating mitochondria membrane stabilization and respiration function. The aim of this study was to define the contribution of STARD7 in the regulation of the intestinal epithelial mitochondrial function and susceptibility to colitis. In silico analyses identified significantly reduced expression of *STARD7* in patients with UC, which was associated with downregulation of metabolic function and a more severe disease phenotype. STARD7 was expressed in intestinal epithelial cells, and STARD7 knockdown resulted in deformed mitochondria and diminished aerobic respiration. Loss of mitochondria function was associated with reduced expression of tight junction proteins and loss of intestinal epithelial barrier integrity that could be recovered by AMPK activation. *Stard7^+/–^* mice were more susceptible to the development of DSS-induced and *Il10^–/–^* spontaneous models of colitis. STARD7 is critical for intestinal epithelial mitochondrial function and barrier integrity, and loss of STARD7 function increases susceptibility to IBD.

## Abstract 2

STARD7 is critical for intestinal epithelial mitochondrial function and barrier integrity and loss of STARD7 function increases susceptibility to IBD.

## Introduction

Inflammatory bowel diseases (IBD), Crohn’s disease (CD), and ulcerative colitis (UC) are chronic inflammatory diseases associated with debilitating symptoms and poor quality of life, and there is a high risk of surgical intervention by 5 years of disease progression (1, 2). Clinical and experimental evidence suggests that the manifestations of IBD result from loss of epithelial barrier integrity and activation of an unrestrained inflammatory response to intestinal microbes in genetically susceptible individuals (3–5). Genome-wide association studies have identified over 200 single nucleotide polymorphisms (SNPs) associated with IBD, and more than 15 of these SNPs are linked to loci associated with epithelial junctional proteins and maintenance of barrier function (6, 7), indicating an important role of intestinal barrier regulation in the pathogenesis of IBD.

Maintenance of intestinal epithelial barrier integrity is an energy-demanding process involving epithelial cell barrier formation and maintenance of complex interactions between epithelial adherens junctional proteins, tight junction proteins, and cytoskeletal elements (8, 9). Disruption of ATP-generating processes in intestinal epithelial cells (IECs) alters homeostasis, including stemness, survival and microbial sensing, and barrier integrity. Colonic biopsy samples from patients with IBD are characterized by diminished ATP levels and cellular stress (10, 11). Furthermore, diminished levels of ATP within the inflamed regions of the colonic mucosa from patients with UC or CD have been associated with downregulation of critical tight junction proteins and reduced intestinal epithelial barrier function (12). Notably, enterocytes from UC have been shown to possess impaired mitochondria with disrupted cristae, which was associated with loss of barrier integrity (13). Collectively, these studies link mitochondrial and intestinal epithelial barrier dysfunction function with IBD.

Mitochondria consist of an outer and inner membrane phospholipid bilayer, which is composed of phosphatidylcholine (PC), phosphatidylethanolamine (PE), cardiolipin (CL), phosphatidylinositol, phosphatidylserine, and phosphatidylglycerol (PG). These phospholipids are essential for mitochondrial membrane structure, function, and energy production (14). While mitochondria possess enzymes to generate CL, PG, and PE, they are incapable of directly synthesizing PC. PC is synthesized in the endoplasmic reticulum (ER) from the substrates, choline and diacylglycerol, via the de novo CDP-choline Kennedy pathway (15). PC is transferred from the ER to cell and organelle membranes via members of the steroidogenic acute regulatory (StAR) protein superfamily including StAR protein-related lipid transfer (START) domain-containing protein (STARD) 2, 7, and 10 (16). STARD7 mRNA encodes a 370–amino acid (43 kDa) precursor protein (STARD7-I) that possesses an N-terminus mitochondria-targeting sequence (MTS) that targets STARD7 to the outer mitochondrial membrane (OMM) and a StAR-related transfer domain that specifically binds to PC and shuttles the phospholipid from the ER to the OMM and inner mitochondrial membrane (IMM) (17–20). STARD7-I also undergoes consecutive proteolytic cleavage by matrix-localized mitochondrial processing peptidase (MPP) and an IMM protease presenilin-associated rhomboid-like (PARL) to generate the mature 33 kDa STARD7 (STARD7-II) that localizes to the IMM, where it participates in shuttling PC from OMM to IMM (19–21). While previous studies have established that STARD7 expression can modulate mitochondrial homeostasis, the contribution of STARD7 to intestinal epithelial mitochondrial function, barrier integrity, and exacerbation of the IBD phenotype remains unknown.

Herein, we show that STARD7 is differentially expressed in patients with UC and CD compared with healthy controls. Patients with STARD7^lo^ UC were characterized by a more severe disease phenotype, and *STARD7* expression was strongly associated with an abnormal metabolic transcriptome. STARD7 knockdown in colonic epithelial cells disrupted mitochondria architecture and respiration and was associated with compromised barrier function. Utilizing STARD7-deficient (*Stard7^+/–^*) mice and employing the DSS-induced and *Il10*^–/–^ spontaneous model of colitis, we found that STARD7 deficiency exacerbated the development of acute and chronic colitis. We show that reconstitution of mitochondrial activity by AMP kinase (AMPK) activation in human intestinal cells led to improved barrier integrity and that treatment of *Stard7^+/–^* with an AMPK agonist demonstrated protection from the colitis phenotype in mice. Collectively, we identify STARD7 as a critical component in ATP-generating processes and maintenance of intestinal epithelial barrier integrity and susceptibility to IBD.

## Results

### Patients with UC are characterized by downregulated Stard7 expression.

**Figure 1:**
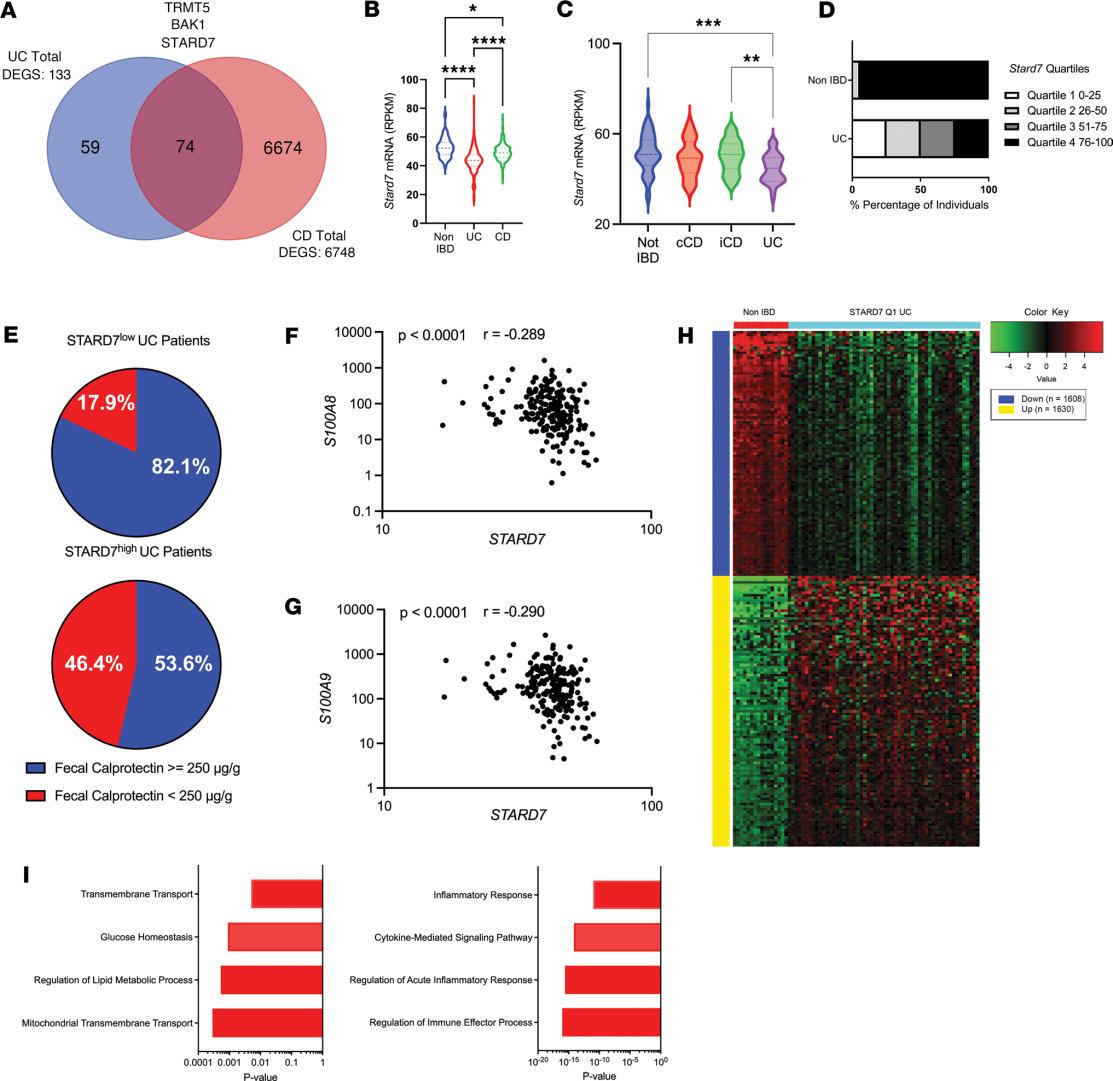
*STARD7* expression is downregulated in IBD. (**A**) Venn diagram of DEGs between UC versus non-IBD and CD versus non-IBD. Select common DEGs are indicated (GSE57945). (**B**) Violin plot showing *STARD7* expression (reads per kilobase per million mapped reads, RPKM) in ileal biopsies from patients with UC, patients with CD, or non-IBD patients (non-IBD *n* = 42; UC *n* = 206, CD *n* = 175). Lines are median and quartiles. (**C**) Violin plot showing *STARD7* expression (RPKM) in rectal biopsies from patients with ileo-colonic CD (iCD), colon-only CD (cCD), or UC or non-IBD patients (non-IBD *n* = 55; UC *n* = 44; iCD *n* = 60; and cCD *n* = 32) (GSE117993). Lines are median and quartiles. (**D**) Stratification of non-IBD and UC cohort based upon *STARD7* expression (quartiles, RPKM) (GSE109142). (**E**) Pie chart indicating the percentage of STARD7^lo^ and STARD7^hi^ UC patients with high levels of fecal calprotectin (≥250 μg/g) 4 weeks after their initial diagnosis. (**F**) Pearson’s coefficient correlation of STARD7 and (**G**) calprotectin (S100A8 and S100A9) mRNA expression in rectal biopsies from UC (GSE109142). (**H**) Heatmap of DEGs based on RNA-Seq data between non-IBD (*n* = 16) and quartile 1 *Stard7* UC (*n* = 56) patients. (**I**) Bar graphs of pathway analysis of upregulated genes (bottom) and downregulated genes (top) in quartile 1 *STARD7* UC patients relative to non-IBD patients, assessed via Gene Ontology (GO) Biological Process Pathways, ranked by *P* value. Data are presented as mean ± SEM. (**B** and **C**) Statistical analysis was performed using 1-way analysis of variance followed by Tukey’s multiple-comparison test or performed using an unpaired *t* test. (**F** and **G**) Correlation analyses were performed using Spearman’s rank correlation coefficient. *P < 0.05, **P < 0.01, ***P < 0.001, ****P < 0.0001.

We examined an RNA-Seq dataset of ileal biopsy samples from a cohort of 322 pediatric patients consisting of treatment-naive CD, treatment-naive UC, and non-IBD individuals (National Center for Biotechnology [NCBI] Gene Expression Omnibus [GEO] GSE57945) (22). We identified 133 differentially expressed genes (DEGs) between UC and non-IBD individuals and 6,748 DEGs between CD and non-IBD individuals (*P* adjusted value < 0.05, Supplemental Table 1, A and B; supplemental material available online with this article; https://doi.org/10.1172/jci.insight.172978DS1). Of the 133 DEGs in UC, 74 of these genes were also differentially expressed in CD (Figure 1A and Supplemental Table 1C). Gene Ontology (GO) pathway analyses of the common IBD transcriptome revealed significant enrichment for genes involved in mitochondrial homeostasis, including *TRMT5*, *BAK1*, and *STARD7* (Figure 1A and Supplemental Table 1D). *STARD7* mRNA expression was significantly more diminished in individuals with UC relative to individuals with CD and the non-IBD control (Figure 1B).

To further explore the relationship between STARD7 expression and IBD, we examined the expression of STARD7 in rectal biopsies from 191 pediatric patients consisting of colon-only CD (cCD), ileo-colonic CD, UC, and non-IBD individuals (GSE117993) (23). In this dataset, all mucosal biopsies were obtained from patients who had macroscopic involvement in the rectal area during endoscopy (23). STARD7 mRNA expression was significantly decreased in rectal biopsies in UC compared with non-IBD (Figure 1C). Furthermore, the levels of STARD7 mRNA expression in UC were significantly decreased compared with ileo-colonic CD (iCD) (Figure 1C). Spearman’s correlation coefficient analyses revealed a negative correlation between STARD7 and S100A9 mRNA expression within UC and not CD, indicating that STARD7 mRNA expression correlates with UC disease severity: UC (*n* = 44), *r* = –0.3481, *P* = 0.022; cCD (*n* = 32), *r* = –0.2757, *P* = 0.1267; and iCD (*n* = 60), *r* = –0.0727, *P* = 0.5829; Spearman’s correlation coefficient (24).

To further explore the differential expression of *STARD7* in UC and the relationship between mucosal inflammation and disease severity, we evaluated a dataset of rectal biopsy samples from a cohort of 226 pediatric individuals, which included treatment-naive UC and non-IBD individuals (GSE109142) (23). Stratification of the UC cohort into quartiles based on *STARD7* mRNA RPKM values — Q1 (0%–25%), 16.78–39.31; Q2 (26%–50%), 39.37–43.81; Q3 (51%–75%), 43.90–48.55, and Q4 (76%–100%), 48.68–64.84 — revealed that UC individuals had the lowest *STARD7* mRNA expression (STARD7^lo^ [STARD7 Q1]) (Figure 1D). Importantly, STARD7^lo^ UC individuals had more severe intestinal inflammation as evidenced by a higher frequency of patients with exaggerated levels of fecal calprotectin (≥250 μg/g) at 4 weeks after their initial diagnosis (Figure 1E). Consistent with this, we observed a negative correlation between the mRNA expression of surrogate markers of clinical disease severity (S100A8 and S100A9) and *STARD7* in rectal biopsies from patients with UC (Figure 1, F and G, and Supplemental Table 2). Further, decreased *STARD7* mRNA expression within individuals with UC was associated with an increase in mRNA expression of *GATA3*, *IFNG*, *IL13*, *IL1B*, *IL23A*, and *IL6*, indicating that *STARD7* expression inversely correlates with mucosal inflammation (Supplemental Table 3). Collectively, these studies suggest that IBD is associated with decreased *STARD7* mRNA expression and levels of *STARD7* expression are associated with the level of disease severity.

To get insight into the pathways associated with altered *STARD7* mRNA expression and the IBD phenotype, we performed pathway enrichment analyses on the DEGs (up and down; 3,238 total number of genes; *P* adjusted value < 0.05) between STARD7^lo^ UC (*n* = 56) and non-IBD patients (*n* = 16) (Figure 1H and Supplemental Table 4). We identified that the downregulated DEGs in the STARD7^lo^ UC transcriptome were enriched for expression of genes associated with mitochondrial transmembrane transport, regulation of lipid metabolic process, glucose homeostasis, and transmembrane transport and upregulated genes were enriched for expression of genes associated with cytokine-mediated signaling pathway and inflammatory responses (Figure 1I and Supplemental Figure 1). Collectively, our in silico studies revealed that *STARD7* mRNA expression is highly diminished in UC patients and suggests a critical role for this lipid binding protein in regulating metabolic processes that can alter inflammatory disease outcomes.

### Loss of STARD7 perturbs intestinal epithelial mitochondrial architecture and function.

**Figure 2:**
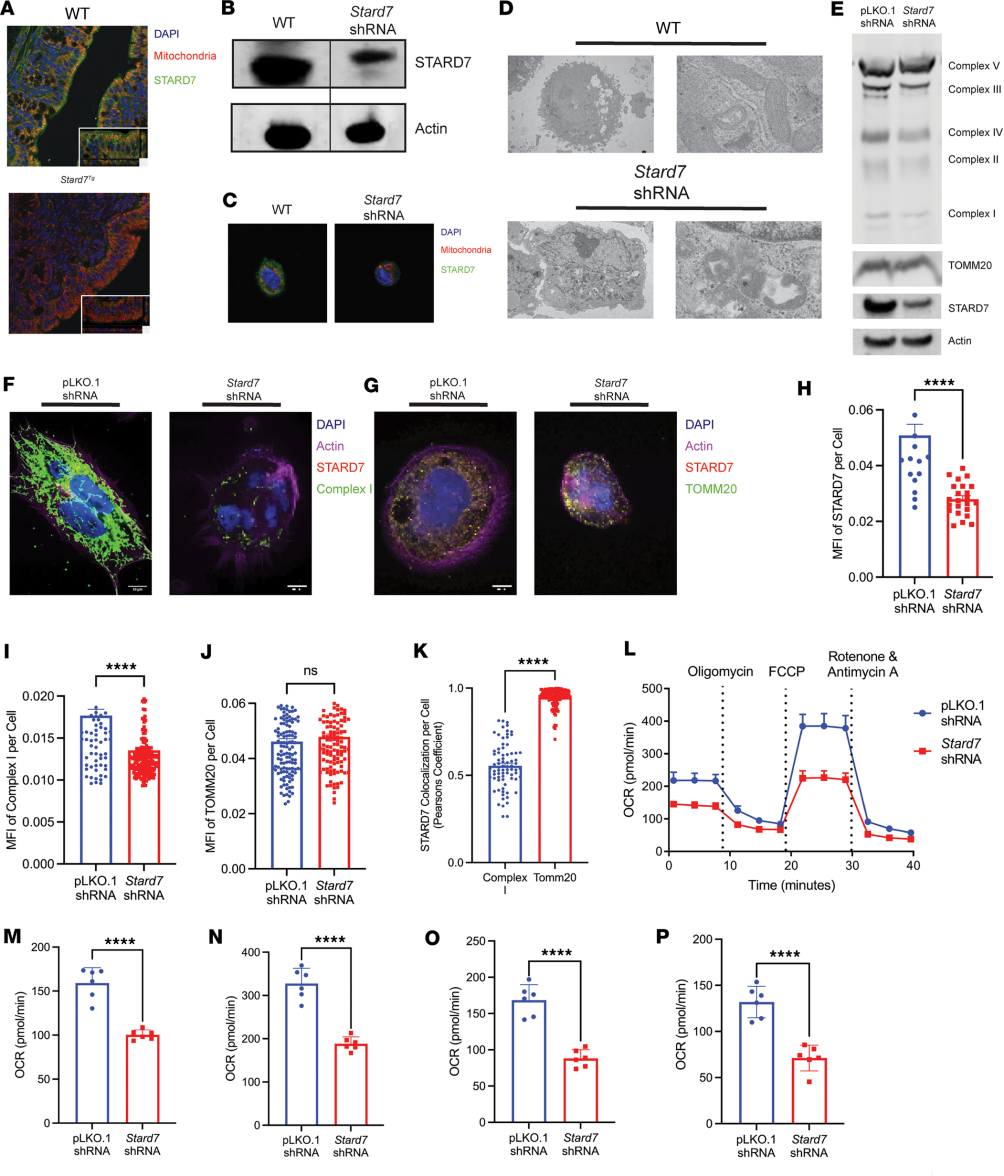
Mitochondrial function is negatively altered in STARD7-deficient colonic epithelial cells. (**A**) Immunofluorescence analysis of STARD7 localization in colonic tissue from WT and *Stard7^+/–^* mice. (**B**) Western blot analyses of STARD7 expression in WT and *Stard7* shRNA–transduced CaCo-2BBe cells. (**C**) Immunofluorescence analysis of STARD7 expression in WT and *Stard7* shRNA–transduced CaCo-2BBe cells. (**D**) Transmission electron microscopy analysis of mitochondrial structure in WT and *Stard7* shRNA–transduced CaCo-2BBe cells. (**E**) Western blot analyses of oxidative phosphorylation proteins in pLKO.1 shRNA– and *Stard7* shRNA–transduced CaCo-2BBe cells. Immunofluorescence analysis of STARD7 expression and either (**F**) complex I or (**G**) TOMM20 expression in pLKO.1 shRNA and *Stard7* shRNA–transduced CaCo-2BBe cells. Quantification (mean fluorescence intensity) of (**H**) STARD7, (**I**) complex I, and (**J**) TOMM20 expression using CellProfiler image analysis software. (**K**) Colocalization analysis between STARD7 and complex I and TOMM20 in pLKO.1 shRNA–transduced CaCo-2BBe cells. (**L**) Seahorse Mito Stress Test was performed on pLKO.1 shRNA– and *Stard7* shRNA–transduced CaCo-2BBe cells where oxygen consumption rate (OCR) was measured over time as cells were exposed at the indicated time points to oligomycin, carbonyl cyanide *p*-trifluoromethoxyphenylhydrazone (FCCP), and rotenone/antimycin A. Measurement of (**M**) basal respiration, (**N**) maximal respiration, (**O**) spare respiratory capacity, and (**P**) ATP production in pLKO.1 shRNA– and *Stard7* shRNA–transduced CaCo-2BBe cells. Data are representative of at least 2 independent experiments with at least 5 replicates per group. Scale bars = 10 µm. Each symbol in **I**–**K** represents an individual cell. Each symbol in **M**–**P** represents an individual well containing 50,000 plated cells. Data are presented as mean ± SEM. Statistical analysis was performed using unpaired *t* test. ****P < 0.0001.

To determine the cellular expression of STARD7, we performed immunofluorescence staining for STARD7 in colonic tissue from WT (C57BL/6) mice and *Stard7^+/–^* (C57BL/6) mice. We show that STARD7 was ubiquitously expressed in IECs, and costaining with a mitochondrial tracker (MitoTracker) localized STARD7 to the mitochondria of colonic epithelial cells (Figure 2A). To determine what intestinal epithelial populations express *STARD7*, we utilized a single-cell RNA-Seq dataset consisting of different colonic epithelial subsets isolated from UC (SCP259) (25). We show *STARD7* expression was enriched in transit-amplifying cells and enterocytes (Supplemental Figure 2, A and B). Remarkably, these *STARD7*^hi^ IEC populations were also enriched for the mitochondrial genes *TOMM20* and *COX14* (Supplemental Figure 2, A and B). Additionally, a discernible reduction in *STARD7*-expressing epithelial cells was observed in the inflamed tissue of patients with UC when the epithelial clusters were compared with samples from healthy individuals (Supplemental Figure 2C). To define the impact of STARD7 deficiency on intestinal epithelial mitochondria function, we utilized lentiviral technology to knock down STARD7 in the colonic epithelial adenocarcinoma cell line (CaCo-2BBe cells). Western blot and immunofluorescence analyses verified that STARD7 shRNA transduction led to knockdown of STARD7 protein (CaCo-2BBe^ΔStard7^) and STARD7 localization to mitochondria in human IECs (Figure 2, B and C). Electron microscopy analyses of CaCo-2BBe cells transduced with control shRNA (CaCo-2BBe^WT^) revealed mitochondria with a large matrix volume between the double membranes and cristae membrane projections into the matrix compartment (Figure 2D). In contrast, CaCo-2BBe^ΔStard7^ mitochondria had altered ultrastructural architecture, including loss of the distinct double membrane structure, a collapsed matrix volume, and dissolution of cristae membrane projections (Figure 2D). Analyses of mitochondrial proteins in CaCo-2BBe^ΔStard7^ cells revealed that loss of STARD7 was associated with substantial reduction in respiratory proteins, such as complex I, complex II, and complex IV, in association with decreased expression of the OMM protein, TOMM20 (Figure 2E). To determine the requirement of STARD7 for mitochondrial network dynamics, we next examined the expression levels of STARD7 and the inner and outer membrane proteins complex I (Figure 2F) and TOMM20 (Figure 2G), respectively. CaCo-2BBe^WT^ cells displayed a robust mitochondrial network consisting of interconnected fused mitochondria with large domains (Figure 2, F and G). In contrast, CaCo-2BBe^ΔStard7^ cells displayed disrupted mitochondrial network with fragmented mitochondria (Figure 2, F and G). Quantification of the mitochondrial network and STARD7 expression revealed significantly reduced expression of STARD7 and complex I in CaCo-2BBe^ΔStard7^ cells compared with CaCo-2BBe^WT^ cells (Figure 2, H and I). Notably, we observed no change in expression of the OMM protein TOMM20, suggesting that reduced STARD7 expression did not influence the number of mitochondria per cell (Figure 2J). Immunofluorescence analyses revealed that STARD7 predominantly localized with TOMM20, suggesting that STARD7 was primarily bound to the OMM (Figure 2K). To a lesser extent, STARD7 also colocalized with the IMM complex I, suggesting that STARD7 can also traffic to this compartment (Figure 2K). To determine whether loss of STARD7 and altered ultrastructural architecture compromised mitochondrial function, we examined glycolytic and oxidative phosphorylation pathways in CaCo-2BBe^WT^ and CaCo-2BBe^ΔStard7^ cells. We show that STARD7 knockdown was associated with severely impaired mitochondrial respiration (Figure 2L), including basal (Figure 2M) and maximal respiration (Figure 2N), in conjunction with decreased spare respiratory capacity (Figure 2O) and ATP production (Figure 2P). This was specific to the oxidative phosphorylation pathway as glycolytic rate was unchanged in CaCo-2BBe^ΔStard7^ cells (Supplemental Figure 3, A and B). In line with this, we also demonstrated that in isolated mitochondria loss of STARD7 impaired oxidative phosphorylation and significantly reduced ATP production (Supplemental Figure 4, A–D). Collectively, our data demonstrate that STARD7 knockdown in IECs alters mitochondria structure and the rate of cellular respiration.

### STARD7 regulates intestinal epithelial barrier integrity.

Mitochondrial function plays an important role in intestinal epithelial function, including maintaining barrier integrity (26–28). To begin to determine if STARD7-mediated mitochondrial dysfunction affected intestinal epithelial barrier integrity, we examined STARD7 and tight junction protein expression in IECs in WT and *Stard7^+/–^* mice. *Stard7^−/−^* mice die embryonically at day 11 while *Stard7^+/–^* mice survive and are fertile (29). Baseline analyses of *Stard7^+/–^* mice revealed no observable differences in gastrointestinal architecture, body weight, or fecal calprotectin levels compared to age- and strain-matched WT mice (Supplemental Figure 5, A–C). Analyses of the fecal microbiome demonstrated no significant difference in the α-diversity of gut microbial communities using the Shannon diversity index (Supplemental Figure 5D). Analyses of the number of operational taxonomic units (OTUs) and community composition (using the Bray-Curtis dissimilarity index) revealed no significant differences between WT and *Stard7^+/–^* mice (Supplemental Figure 5, E and F). Principal component analysis and ordination with permutational multivariate analysis of variance revealed no significant difference in community composition across WT and *Stard7^+/–^* mice (Supplemental Figure 5G). The most abundant taxa in each group at the OTU level are ranked, displaying overlap in prevalent OTUs (Supplemental Figure 5H).

**Figure 3:**
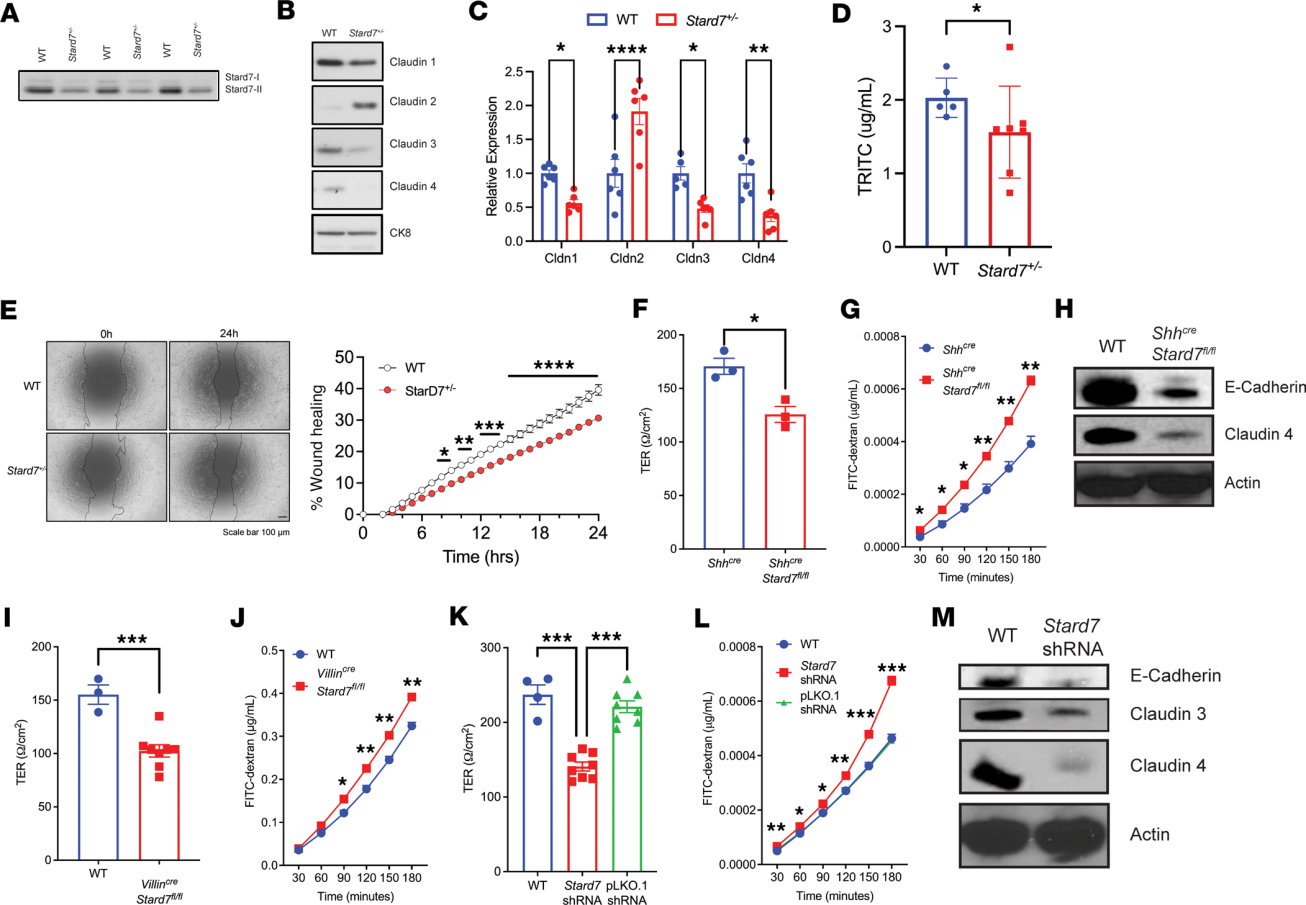
STARD7 deficiency alters homeostatic intestinal epithelial barrier function. Western blot of (**A**) STARD7 and (**B**) tight junction proteins in WT and *Stard7^+/–^* epithelial cells. (**C**) Densitometry analyses of tight junction proteins in WT and *Stard7^+/–^* epithelial cells (WT *n* = 6; *Stard7^+/–^* *n* = 6). (**D**) In vivo ileal epithelial permeability to FITC-dextran in WT and *Stard7^+/–^* mice (*n* = 6 WT; *n* = *8* *Stard7^+/–^* mice). (**E**) Representative photomicrograph (left) and quantification (right) of wound repair in primary epithelial monolayers from WT and *Stard7^+/–^* mice. Scale bars = 100 μm. Dashed line, edges of wounds. (**F**) Transepithelial electric resistance in *Shh^cre^* *Stard7^fl/fl^* epithelial cells (*Shh^cre^* *n* = 3; *Shh^cre^* *Stard7^fl/fl^* *n* = 3). (**G**) Time course of FITC-dextran flux in *Shh^cre^* *Stard7^fl/fl^* epithelial cells (*Shh^cre^* *n* = 3; *Shh^cre^* *Stard7^fl/fl^* *n* = 3). (**H**) Western blot of tight junction proteins in WT and *Shh^cre^* *Stard7^fl/fl^* epithelial cells. (**I**) Transepithelial electric resistance in *Villin^cre^* *Stard7^fl/fl^* epithelial cells (WT *n* = 3; *Villin^cre^* *Stard7^fl/fl^* *n* = 8). (**J**) Time course of FITC-dextran flux in *Villin^cre^* *Stard7^fl/fl^* epithelial cells (WT *n* = 3; *Villin^cre^* *Stard7^fl/fl^* *n* = 3). (**K**) Transepithelial electric resistance in WT, pLKO.1 shRNA–transduced and *Stard7* shRNA–transduced CaCo-2BBe cell monolayers (WT *n* = 4; pLKO.1 *n* = 8; *Stard7* *n* = 8). (**L**) Time course of FITC-dextran flux in WT and *Stard7* shRNA–transduced CaCo-2BBe cell monolayers (WT *n* = 4; pLKO.1 *n* = 4; *Stard7* *n* = 4). (**M**) Western blot of tight junction proteins in WT and *Stard7* shRNA–transduced CaCo-2BBe cells. Data are representative of at least 2 independent experiments with at least 3 replicates per group. Each symbol in **F**, **G**, **I**, and **K** represents an individual well containing 500,000 cells. Data are presented as mean ± SEM. Statistical analysis performed using 1-way analysis of variance followed by Tukey’s multiple-comparison test or performed using an unpaired *t* test. *P <0.05, **P <0.01, ***P <0.001, ****P <0.0001.

Analyses of the intestinal epithelial compartment revealed that haplotypic expression of STARD7 in colonic IECs led to a reduction in both STARD7-I and STARD7-II protein and that this associated with markedly altered IEC tight junction proteins (Figure 3, A and B). Notably, IECs from *Stard7^+/–^* mice had decreased Claudin-1, Claudin-3, and Claudin-4 and increased Claudin-2 expression compared with WT IECs (Figure 3, B and C). Consistent with the diminution of junctional proteins in the IECs, performing an in vivo ileal epithelial permeability assay, we show that haplotypic STARD7 expression resulted in a significant increase in FITC-dextran flux in *Stard7^+/–^* mice compared with WT mice, indicating a nonredundant and critical role for STARD7 in epithelial barrier function in vivo (Figure 3D). The demonstrated decrease in expression of the junctional proteins Claudin-2 and Claudin-4 would also predict a defect in IEC wound healing responses. To test this, we generated 2D epithelial cell monolayers from WT and *Stard7^+/–^* colonoids and performed scratch wound healing assays. Time lapse imaging of scratch-induced wounds revealed significantly delayed closure in *Stard7^+/–^* intestinal epithelial monolayers compared with WT epithelial monolayers (*P* < 0.0001, Figure 3E). Collectively, these studies show that haplotypic STARD7 expression in IECs alters intestinal epithelial barrier integrity and wound healing responses.

To identify if the STARD7 defects were intrinsic to the epithelial compartment, we crossed *Stard7^fl/fl^* mice onto the *Shh^cre^* mouse background to delete STARD7 from the epithelial compartment. Ex vivo analyses of the colonic mucosa of *Shh^cre^* *Stard7^WT^* (WT) and *Shh^cre^* *Stard7^fl/fl^* mice revealed that STARD7 deletion in epithelial cells resulted in a significant decrease in transepithelial electrical resistance (TER) and increased FITC-dextran flux relative to WT (*Shh^cre^*) epithelial cells (Figure 3, F and G). The diminished colonic epithelial barrier function was associated with dysregulated expression of the adherens junctional and tight junction proteins E-cadherin and Claudin-4, respectively (Figure 3H). To verify intestinal epithelial involvement, we backcrossed *Stard7^fl/fl^* mice with *Villin^cre^* mice and revealed that deletion of STARD7 in the intestinal epithelial compartment led to decreased TER and increased FITC-dextran flux across the mucosa of *Villin^cre^* *Stard7^fl/fl^* mice compared with WT mice (Figure 3, I and J). Consistent with our ex vivo findings, STARD7 knockdown in CaCo-2BBe cells exhibited a significant reduction in TER and FITC-dextran flux (Figure 3, K and L). In addition, this was associated with decreased expression of E-cadherin, Claudin-3, and Claudin-4 (Figure 3M). Collectively, our data reveal that loss of STARD7 within the epithelial compartment diminished epithelial junctional protein expression and epithelial barrier integrity.

### AMPK agonists reconstitute mitochondria ultrastructure, architecture, and barrier integrity in STARD7-deficient epithelial cells.

**Figure 4:**
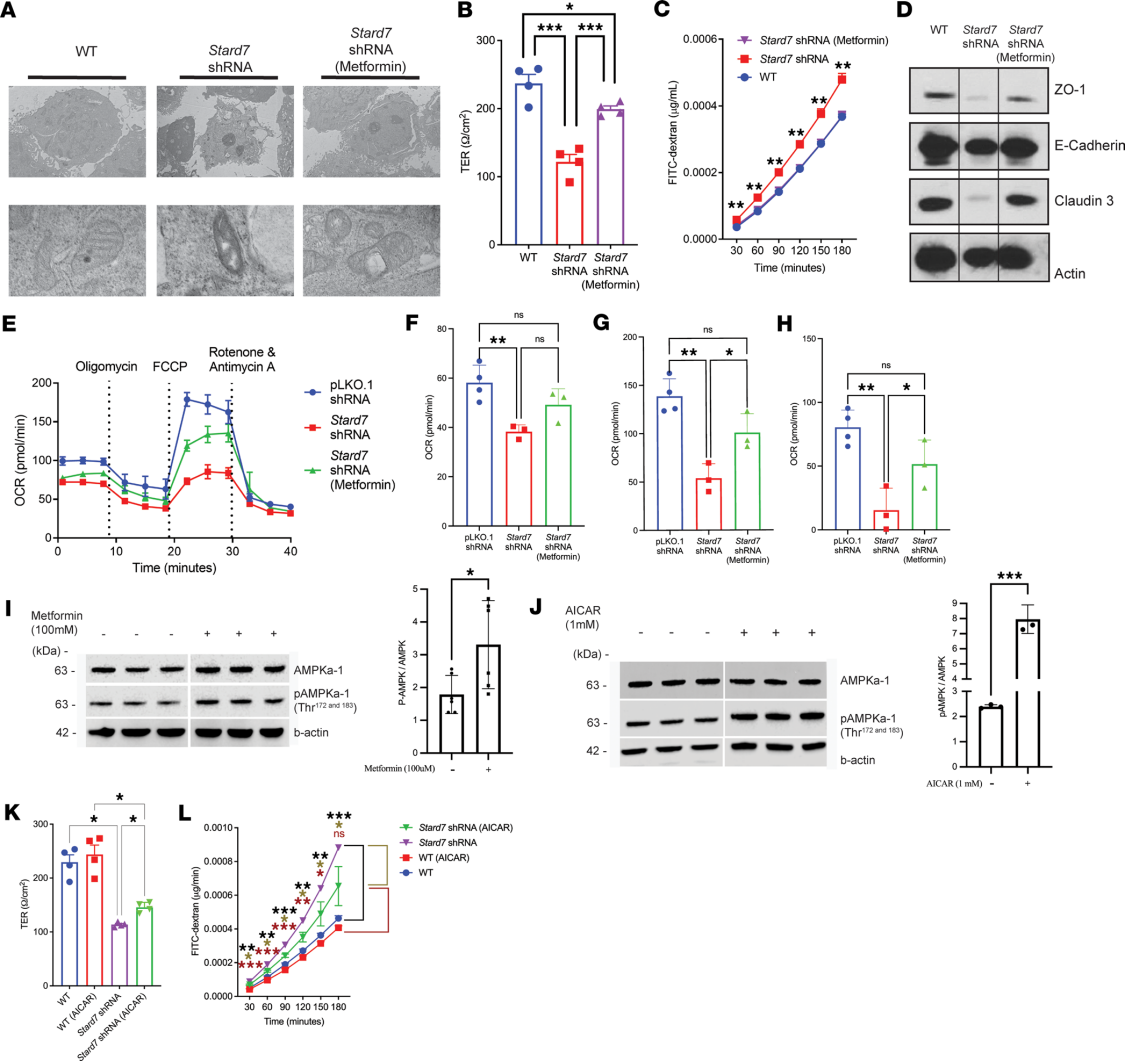
AMPK agonists reconstitute mitochondria ultrastructure, architecture, function, and barrier integrity in STARD7-transduced IECs. (**A**) Electron microscopy of WT, *Stard7* shRNA–transduced, and metformin-stimulated cells. (**B**) Transepithelial electric resistance in WT, *Stard7* shRNA–transduced, and metformin-stimulated CaCo-2BBe cell monolayers (WT *n* = 4; *Stard7* *n* = 4; metformin-stimulated *Stard7* *n* = 4). (**C**) FITC-dextran flux analyses in WT, *Stard7* shRNA–transduced and metformin-stimulated CaCo-2BBe cell monolayers (WT *n* = 4; *Stard7* *n* = 4; metformin-stimulated *Stard7* *n* = 4). (**D**) Western blot of tight junction proteins in WT, *Stard7* shRNA–transduced, and metformin-stimulated *Stard7* shRNA–transduced CaCo-2BBe cells. (**E**) Seahorse Mito Stress Test was performed on pLKO.1 shRNA–, *Stard7* shRNA–, and metformin-pretreated *Stard7* shRNA–transduced CaCo-2BBe cells where OCR was measured over time as cells were exposed at the indicated time points to oligomycin, FCCP, and rotenone/antimycin A. Measurement of (**F**) basal respiration, (**G**) maximal respiration, and (**H**) spare respiratory capacity in pLKO.1 shRNA–, *Stard7* shRNA–, and metformin-pretreated *Stard7* shRNA–transduced CaCo-2BBe cells. Representative Western blot (left panel) and quantification (right panel) of total AMPKα-1, phosphorylated AMPKα-1 (Thr^172^ ^and^ ^183^) and β-actin in (**I**) metformin-stimulated (100 μM) and (**J**) AICAR-stimulated (1 μM) WT CaCo-2BBe cells (48 hours). Lanes were run on the same gel but were noncontiguous. Transepithelial electric resistance (**K**) and time course of FITC-dextran flux (**L**) in WT and *Stard7* shRNA–transduced CaCo-2BBe cell monolayers following vehicle or AICAR (1 μM) stimulation (*n* = 4 per group). Data are representative of at least 3 independent experiments with at least 3 replicates per group. Each symbol in **B** and **K** represent an individual well containing 500,000 plated cells. Each symbol in **F**–**H** represent an individual well containing 50,000 plated cells. Data are presented as mean ± SEM. Statistical analysis was performed using 1-way analysis of variance followed by Tukey’s multiple-comparison test or performed using an unpaired *t* test. *P < 0.05, **P < 0.01, ***P < 0.001.

Dysregulated mitochondrial respiration leads to activation of protection mechanisms such as increased AMPK activity that in turn upregulates mitochondrial biogenesis and respiration (30, 31) and enhances epithelial barrier integrity (32, 33). To test the involvement of mitochondrial stress induced by STARD7 deficiency in intestinal epithelial barrier dysfunction, CaCo-2BBe^ΔStard7^ monolayers were stimulated with the AMPK activator, metformin, and mitochondrial structure and barrier function were assessed. Metformin treatment was sufficient to recover mitochondrial architecture, including reconstitution of matrix volume and long cristae membrane projections, in CaCo-2BBe^ΔStard7^ cells (Figure 4A). Concurrently, metformin improved intestinal epithelial barrier function; metformin-treated CaCo-2BBe^ΔStard7^ cells demonstrated comparable levels of barrier integrity to those of WT epithelial cells (Figure 4, B and C). The strengthened barrier was associated with increased expression of tight junction proteins including zonula occludens-1 (ZO-1) and Claudin-3 (Figure 4D). Metformin exposure of CaCo-2BBe^ΔStard7^ cells also led to improved mitochondrial respiration (Figure 4E), including basal (Figure 4F) and maximal respiration (Figure 4G), and spare respiratory capacity (Figure 4H). Metformin has pleiotropic actions and can act via AMPK-dependent and -independent mechanisms. To test whether activation of AMPK can reconstitute the barrier integrity in CaCo-2BBe^ΔStard7^ cells, we used the AMPK-selective agonist 5-aminoimidazole-4-carboxamide-1-β-d-ribofuranoside (AICAR) (34). We show that stimulation of CaCo-2BBe cells with metformin or AICAR induced AMPK activation as evidenced by phosphorylation of Thr-172 and Thr-183 (Figure 4, I and J). Notably, treatment with AICAR led to improved intestinal epithelial barrier integrity (Figure 4, K and L). Cumulatively, our results demonstrate that activation of the AMPK pathway can rescue mitochondrial dysfunction and epithelial barrier function induced by STARD7 deficiency.

### STARD7 deficiency increased susceptibility to both innate immune cell– and T cell–dependent colitis.

**Figure 5:**
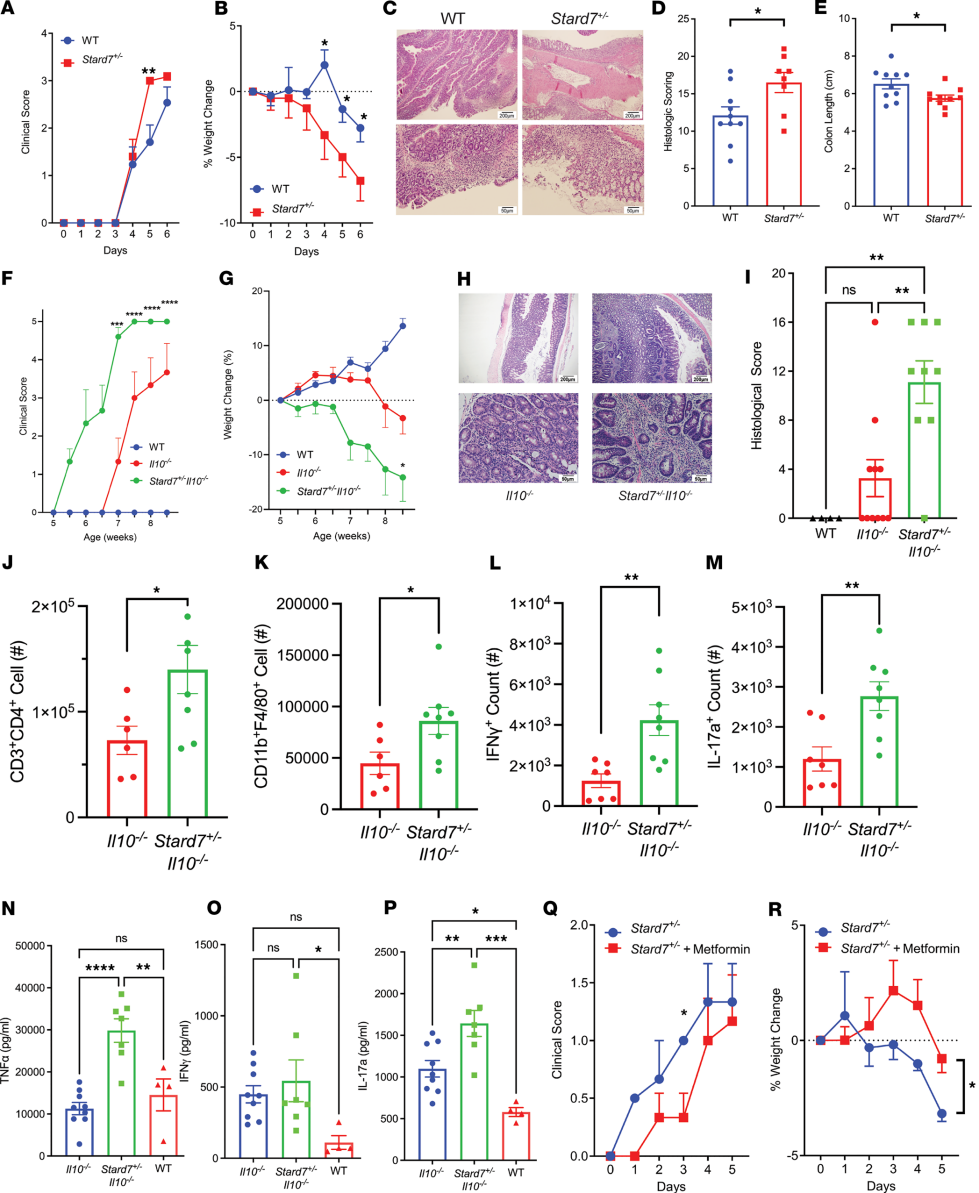
Loss of *Stard7* exaggerates the development of colitis. (**A**) Clinical score, (**B**) percentage weight change, and (**C**) representative image of colon histology from WT and *Stard7^+/–^* mice receiving 2.5% DSS (WT *n* = 17; *Stard7^+/–^* *n* = 15). (**D**) Histological scoring from WT and *Stard7^+/–^* mice at day 7 of DSS exposure (WT *n* = 10; *Stard7^+/–^* *n* = 9). (**E**) Colon lengths from WT and *Stard7^+/–^* mice (WT *n* = 10; *Stard7^+/–^* *n* = 10). (**F**) Clinical score, (**G**) percentage weight change, and (**H**) representative image of colon histology from *Il10^–/–^* and *Stard7^+/–^* *Il10^–/–^* mice (WT *n* = 4; *Il10^–/–^* *n* = 7; *Stard7^+/–^* *Il10^–/–^* *n* = 8). (**I**) Histological scoring from *Il10^–/–^* and *Stard7^+/–^* *Il10^–/–^* mice (*Il10^–/–^* *n* = 7; *Stard7^+/–^* *Il10^–/–^* *n* = 8). (**J**) Counts (#) of CD3^+^CD4^+^ T cells in the mLNs of mice (*Il10^–/–^* *n* = 7; *Stard7^+/–^* *Il10^–/–^* *n* = 8). (**K**) Counts (#) of CD11b^+^F4/80^+^ macrophages in the mLNs of mice (*Il10^–/–^* *n* = 7; *Stard7^+/–^* *Il10^–/–^* *n* = 8). Counts (#) of (**L**) CD4^+^IFN-γ^+^ and (**M**) CD4^+^IL-17a^+^ T cells in the mLNs of mice (*Il10^–/–^* *n* = 7; *Stard7^+/–^* *Il10^–/–^* *n* = 8). Systemic levels of (**N**) TNF-α, (**O**) IFN-γ, and (**P**) IL-17a in serum of colitis mice (*Il10^–/–^* *n* = 7; *Stard7^+/–^* *Il10^–/–^* *n* = 8). (**Q**) Clinical score (*Stard7^+/–^* *n* = 3, metformin-treated *Stard7^+/–^* *n* = 6) and (**R**) percentage weight change (*Stard7^+/–^* *n* = 3, metformin-treated *Stard7^+/–^* *n* = 6) of *Stard7^+/–^* mice receiving 2.5% DSS. Top row magnification = 4×, bottom row magnification 20×, 4× scale bar represents 200 μm, 20× scale bar represents 50 μm. Data encompass 3 independent experiments. Each symbol in **D**, **E**, and **I**–**P** represent an individual mouse. Data are presented as mean ± SEM. Statistical analysis was performed using 1-way analysis of variance followed by Tukey’s multiple-comparison test or performed using an unpaired *t* test. *P < 0.05, **P < 0.01, ***P < 0.001, ****P < 0.0001.

Next, we tested the role of STARD7 in the innate immune cell–driven colitis phenotype. WT (C57BL/6) and *Stard7^+/–^* mice were mock-treated or treated with DSS and monitored for disease development over the course of 6 days. DSS exposure of WT mice induced colitis symptoms by day 4, including dehydration, bloody stool, and rectal prolapse that got progressively worse throughout the duration of the experiment (Figure 5A). These symptoms were associated with a loss in body weight from day 5 of DSS exposure (Figure 5B). Histological analysis of the colonic tissue from WT mice showed substantial intestinal epithelial injury, including epithelial apoptosis, villus atrophy, and inflammatory infiltrates (Figure 5, C and D). DSS treatment of *Stard7^+/–^* mice led to an exaggerated colitis phenotype, including increased loss of body weight, colon shortening, and histopathological changes to the colon, including severe transmural infiltration and epithelial destruction (Figure 5, A–E).

Next, we interrogated the role of STARD7 in the T cell–driven colitis phenotype. *Stard7^+/–^* mice were mated with *Il10^–/–^* mice on C57BL/6 background and monitored for the development of the spontaneous colitis phenotype (35). *Il10^–/–^* mice demonstrated the spontaneous colitis phenotype as evidenced by decreased activity, diarrhea, excreted perianal mucus, dehydration, and diminished weight gain at 7 weeks of age (Figure 5, F and G). Histological analysis of the colon from *Il10^–/–^* mice showed evidence of epithelial erosion and inflammation (Figure 5, H and I). *Stard7^+/–^* *Il10^–/–^* mice exhibited more severe symptoms of colitis and demonstrated exaggerated weight loss before they reached 5–6 weeks of age, indicating that the STARD7 deficiency was associated with early-onset colitis development (Figure 5, F and G). Congruently, histological analyses revealed that the colon of *Stard7^+/–^* *Il10^–/–^* mice was characterized by increased transmural inflammation and crypt destruction relative to *Il10^–/–^* mice (Figure 5, H and I). Consistent with increased inflammation, we observed increased cell number of CD3^+^CD4^+^ T cells and CD11b^+^F4/80^+^ myeloid populations in the mesenteric lymph nodes (mLNs) of *Stard7^+/–^* *Il10^–/–^* mice compared with *Il10^–/–^* mice (Figure 5, J and K). Additionally, the frequency of CD4^+^IFN-γ^+^ T cells and CD4^+^IL-17a^+^ T cells was significantly enhanced in *Stard7^+/–^* *Il10^–/–^* mice (Figure 5, L and M). Last, *Stard7^+/–^* *Il10^–/–^* mice were marked by increased levels of systemic TNF-α and IL-17a but not IFN-γ (Figure 5, N–P). Together, these data show that mice deficient in STARD7 have increased susceptibility to development of both innate and T cell–dependent colitis. To test whether metformin could abrogate the exaggerated colitis phenotype observed in *Stard7^+/–^* mice, we pretreated *Stard7^+/–^* mice with metformin and examined DSS-induced colitis outcomes. Pretreatment of *Stard7^+/–^* mice with metformin significantly delayed the onset of DSS-induced colitis, as evidenced by delayed development of clinical symptoms and diminished weight loss (Figure 5, Q and R). Together, these data show that mice with diminished STARD7 expression have increased susceptibility to development of both innate and T cell–dependent colitis and that metformin treatment can abrogate the exaggerated colitis phenotype.

## Discussion

Herein, we demonstrate 1) differential expression of the mitochondrial homeostasis gene STARD7 in both CD and UC and that STARD7 mRNA expression inversely correlated with disease severity in UC; 2) STARD7 was localized to the mitochondria of IECs; 3) STARD7 regulates intestinal epithelial mitochondrial dynamics and that loss of STARD7 results in altered ultrastructural architecture and mitochondrial stress; 4) STARD7-mediated mitochondria dysfunction in IECs was associated with loss of integrity of the intestinal barrier, resulting in increased susceptibility to colitis; and 5) metformin treatment overcame STARD7 deficiency and reconstituted mitochondrial ultrastructure and function and intestinal epithelial barrier function and protected from the colitis phenotype.

Previous studies have reported that STARD7 is recruited to the mitochondria via an MTS, where it anchors to the OMM (19). Consistent with this, we show that STARD7 was predominately localized to the OMM of mitochondrial IECs. While the mechanism of transfer of PC by STARD7-I to OMM is not fully elucidated, it is speculated that the anchoring of STARD7-I to the outer leaflet of the OMM positions the cytoplasmic facing C-terminal START domain of STARD7 in close proximity to PC anchored at the cytoplasmic surface of the ER membranes, which are held via ER-mitochondria contacts (19). STARD7-I is thought to bind to ER-bound PC, leading to a conformational change and the transfer of PC to the OMM at these organelles contact sites. We also observed STARD7 in association with the IMM of IECs, suggesting that STARD7 can also shuttle PC to the IMM. Consistent with this, Saita et al. recently demonstrated that STARD7-I translocates across the OMM via the translocase of the outer membrane complex localizing to the mitochondria intramembrane space. There STARD7-I undergoes stepwise proteolytic cleavage mediated by MPPs and PARL, generating the mature form STARD7-II, which embeds within the IMM, where it transfers PC from the OMM to the IMM (19–21). Investigators have demonstrated 1) that loss of STARD7 decreases the PC content specifically in the IMM but not in the OMM, without significantly affecting the levels of other phospholipids (21), and 2) that reconstitution of STARD7-I and not STARD7-II is sufficient to rescue the STARD7-deficient mitochondrial abnormalities (36). Collectively, these studies indicate that STARD7 is primarily an intramitochondrial lipid transfer protein involved in shuttling PC between the OMM and IMM.

We show that knockdown of STARD7 led to altered ultrastructural architecture, including dissolution of cristae membrane projections and reduced intramembrane matrix volumes. Why STARD7 knockdown results in disruption of altered ultrastructure architecture remains unclear. However, this is likely related to the integral role PC plays in maintaining the formation and fluidity of mitochondrial membranes, membrane protein stabilization, and cristae shape dynamics (37, 38). Loss of STARD7 and diminution of PC levels within the OMM and IMM is likely to disrupt the mitochondrial membrane morphology and organization, causing altered cristae shape dynamics. Indeed, we show that diminished STARD7 in IECs led to cristae malformation, and this was associated with marked loss of IMM folds and a concomitant decrease in mitochondrial complexes I, II, and III resulting in diminished mitochondrial respiration.

We also show that STARD7 deficiency in IECs was associated with loss of integrity of the intestinal epithelial barrier. We found that the reduced intestinal epithelial barrier integrity was associated with disrupted expression of colonic intestinal epithelial tight junction proteins including Claudin-3 and Claudin-4 and increased expression of pore-forming Claudin-2. Claudin-3 and Claudin-4 have been shown to be highly expressed within the colonic intestinal epithelia and act as sealing components of the tight junction and strengthen paracellular permeability (39). Genetic abrogation of Claudin-3 and Claudin-4 has been associated with increased permeability, and conversely, overexpression of Claudin-3 led to improved epithelial permeability (40–42). Our demonstration that AMPK activation led to restitution of mitochondria architecture, increased Claudin-3 expression, and improved intestinal epithelial barrier function suggests that STARD7-mediated dysregulation of mitochondrial function is responsible for the altered intestinal epithelial barrier properties. We speculate that loss of STARD7 leads to reduced mitochondrial respiratory function, which impairs IEC barrier gene expression and decreases barrier function. Consistent with this, ammonia-induced mitochondrial dysfunction in IECs has been associated with increased epithelial permeability and reduced the membrane localization of barrier-forming proteins ZO-1, ZO-2, and Claudin-3 (43). In epithelial cells, RhoA GTPase–dependent formation of tight junctions and IEC polarization are regulated by ATP-dependent Na^+^K^+^ ATPase (34, 44, 45). Thus, STARD7 is uniquely poised to modulate IEC barrier properties through regulation of IEC energy requirements (ATP levels).

STARD7 haploinsufficiency in mice was associated with increased susceptibility to innate immune cell– and T cell–dependent colitis. Recently there have been several studies demonstrating that compromised intestinal epithelial mitochondrial function can alter colitis susceptibility. The PPARγ coactivator 1α (PGC1α) is a protein highly expressed within the intestinal epithelial layer and a key regulator of mitochondrial biogenesis. Targeted disruption of PGC1α within the intestinal epithelial compartment of mice promoted protection from DSS colitis (46). Intestinal epithelial deletion of prohibitin 1, an IMM protein that is critical for optimal respiratory chain assembly and function and maintenance of Paneth cells (47, 48), leads to IEC mitochondrial dysfunction and development of spontaneous ileal inflammation (49). Similar to our observations, reconstitution of mitochondria function was associated with amelioration of susceptibility to the colitis phenotype (49). Finally, disruption of succinate dehydrogenase A, a key component of mitochondrial complex II within IECs, also led to altered epithelial bioenergetics, increased ROS formation, and T cell–mediated immunopathology (50). Collectively, these studies show that compromise of mitochondrial integrity and function in IECs leads to detrimental changes of essential mitochondrial structure and reduction in bioenergetics, which alter epithelial barrier function and colitis susceptibility. We show that AMPK activation could compensate for loss of STARD7 and reconstitute mitochondrial architecture, expression of junctional proteins, and barrier permeability. This effect was associated with delaying the onset of DSS-induced colitis in STARD7-deficient mice. Metformin is known to activate AMPK (51), which stimulates mitochondrial fission (52) and mitochondrial biogenesis under stress conditions (30, 31). Furthermore, AMPK is also known to have a beneficial impact on the formation of tight junction proteins and strengthening of the intestinal barrier (53, 54). Consistent with this, we show that metformin and AMPK selective agonist AICAR stimulation of STARD7-knockdown IECs enhanced intestinal epithelial barrier properties. These studies support the notion that STARD7 dysregulation of mitochondria bioenergetics leads to disruption of the intestinal epithelial barrier and increased susceptibility to colitis.

The decreased expression of STARD7 in mucosal biopsy samples from patients with IBD and the negative correlation between STARD7 and proinflammatory cytokine mRNA expression suggests that inflammatory mediators may regulate STARD7 mRNA expression. To test whether IBD-relevant cytokines including IL-1β, IL-6, and TNF-α alter STARD7 mRNA expression in IECs, we stimulated CaCo-2BBe cells with the cytokines IL-1β, IL-6, and TNF-α for 6 and 24 hours and measured STARD7 mRNA expression. We show that the cytokines did not alter STARD7 mRNA expression in IECs (Supplemental Figure 6). This is consistent with recent studies utilizing primary human colonoids showing that TNF-α, IFN-γ, IL-17A, and IL-22 stimulation of human colonoids did not alter STARD7 mRNA expression (4). A recent study examining the effect of hypoxia and differentiation on human intestinal organoids (HIOs’) gene expression demonstrated that differentiation of HIOs promotes a significant increase in *STARD7* mRNA expression. HIO differentiation was induced by decreasing the levels of the Notch signaling regulatory molecule, Wnt-3A (55). Notch signaling is known to suppress transcription factor 4 (TCF4)/β-catenin target genes (56), and TCF4 and the β-catenin coactivator glycogen synthase kinase-3β regulate human STARD7 transcription (57). β-Catenin/TCF4 bind to a TCF4 consensus binding site –614/–608 bp 5′ StarD7 ATG start site, and deletion of this region inhibits STARD7 transcription in choriocarcinoma JEG-3 cells (58).

There is growing clinical evidence suggesting that mitochondrial dysfunction contributes to induction of IBD (59, 60). Approximately 5% of IBD genetic susceptibility factors that have been identified are involved in the regulation of mitochondrial homeostasis, including SLC25A28 encoding mitoferrin 2, PARK7 encoding Parkinson’s disease 7, VARS encoding valine-tRNA ligase, and RNF5 encoding E3 ubiquitin-protein ligase RNF5 (61–63). SLC25A28 is an iron transporter that is localized to the IMM and is important for mitochondrial iron uptake (64, 65). Notably, knockdown of SLC25A28 leads to a decrease in mitochondrial Fe^2+^ uptake, dysregulation of mitochondrial depolarization, and decrease in intracellular ATP level and mitochondrial dysfunction (65–67). Given the known importance of PC in establishing IMM morphology (68) and subsequent assembly of the electron transport chain and the proton gradient, loss of STARD7 function would be anticipated to have a similar outcome. Interestingly, we observed a stronger link between altered STARD7 expression and UC than CD. This is consistent with previous reports demonstrating reduced expression of mitochondrial DNA-encoded genes in UC and not CD (23). We show that metformin treatment in mice can alleviate some of the dysregulation of mitochondria bioenergetics and improve intestinal epithelial barrier properties and reduce susceptibility to colitis. Notably, metformin treatment is associated with improved IBD outcomes and decreased need for oral and intravenous steroids in UC patients with comorbid type 2 diabetes mellitus (69).

In conclusion, we show that STARD7 expression in colonic epithelial cells is important for mitochondrial homeostasis and energy production and diminution of STARD7 expression leads to the reduction in mitochondria function, resulting in a decrease in intestinal epithelial barrier integrity and enhanced susceptibility to colitis. Furthermore, we show that treatment with AMPK agonists can recover mitochondrial function, reconstitute intestinal epithelial barrier integrity, and reduce colitis susceptibility. These studies support the emerging concept that underlying mitochondrial dysfunction contributes to induction of IBD, particularly UC, and that targeting of mitochondrial associated networks is a therapeutic modality for treatment and prevention of IBD.

## Methods

### Sex as a biological variable.

Sex was not considered as a biological variable; both female and male mice were used.

### Mice.

C57BL/6 (5–10 weeks old) male and female mice were used in this study. *Il10^–/–^* mice (B6.129P2-Il10tm1Cgn/J), *Villin^cre^* mice [B6.Cg-Tg(Vil1-cre)997Gum/J], and *Shh^cre^* mice [Shh^tm1(EGFP/Cre)^] (C57BL/6J) were originally obtained from Jackson Laboratory. *Stard7^+/–^* (C57BL/6J) and *Stard7^fl/fl^* (C57BL/6J) were provided by Timothy Weaver, Cincinnati Children’s Hospital Medical Center, Cincinnati, Ohio, USA (29, 70). To generate *Stard7^+/–^* *Il10^–/–^* mice (C57BL/6J), the *Stard7^+/–^* mice were intercrossed with *Il10^–/–^* mice. C57BL/6 strain purity was confirmed (>99%) by DartMouse congenic analyses. All mouse strains were cohoused.

### Experimental colitis models.

DSS (ICN Biomedical Inc.) was supplied in the drinking water as a 2.5% (w/v) solution. Euthanasia and postmortem analyses were performed 6 days after treatment with DSS. Clinical disease was scored based on prior descriptions (71). For the *Il10^–/–^* spontaneous model of colitis, cohoused *Il10^–/–^* and *Stard7^+/–^* *Il10^–/–^* mice were monitored for colitis development from 5 weeks of age. Euthanasia and postmortem analyses were performed at 10 weeks of age. Body weight of colitis mice was measured twice a week, and lymph nodes, small intestine, and colonic tissue were collected for analysis at 10 weeks. Weight changes were calculated as a percentage of the starting body weight (day 0). Clinical disease was scored based on prior descriptions. In some experiments metformin was administered 48 hours and 24 hours prior to and during the consecutive days of DSS exposure. Mice received by oral gavage vehicle (PBS) or metformin (500 mg/kg; PBS from Cayman Chemicals) daily, and colitis outcome parameters were measured as described (71). For all experiments, mice that lost at least 20% of their body weight were euthanized in accordance with IACUC protocols.

### Histology.

Harvested tissues were washed with PBS, then fixed flat or Swiss rolled overnight in 4% paraformaldehyde. Paraffin-embedded tissues were stained with hematoxylin and eosin and analyzed by bright-field microscopy. Histological scoring was performed on ascending, transverse, and descending colon and rectum segments as previously described (71).

### Western blot.

IECs were lysed in protein lysis buffer (10% glycerol, 20 mM Tris HCl [pH 7], 137 mM NaCl, 2 mM EDTA, and 1% NP-40) supplemented with a proteinase inhibitor cocktail (Thermo Fisher Scientific) and PhoSTOP phosphatase inhibitors (Roche). Protein lysates were cleared of insoluble material through centrifugation at 2,000*g*, and the resulting protein lysates were subjected to SDS-PAGE. Proteins were wet transferred to 0.2 mm nitrocellulose membranes (Thermo Fisher Scientific), which were blocked using 3% BSA in 1% TBS with Tween 20 (TBST) buffer for 1 hour at room temperature. Membranes were incubated overnight using the following primary antibodies: α–β-actin (13E5, Cell Signaling Technology), α-STARD7 (PA5-30772, Thermo Fisher Scientific), α–Claudin-1 (MH25, Thermo Fisher Scientific), α–Claudin-2 (12H12, Thermo Fisher Scientific), α–Claudin-3 (OTIIE7, Novus), α–Claudin-4 (ZMD.306, Thermo Fisher Scientific), α–E-Cadherin (AF648, R&D Systems, Bio-Techne), α-CK8 (EP1628Y, Thermo Fisher Scientific), α-TOMM20 (EPR15581-54, Abcam), and Oxphos WB cocktail (Abcam). Primary antibodies were used at 1:500, 1:1,000, or 1:2,000 dilutions in blocking buffer. Membranes were washed in TBST and incubated with the secondary antibody (goat α–rabbit-HRP or goat α–mouse-HRP) (31460, Thermo Fisher Scientific, and 31430, Thermo Fisher Scientific, respectively) at a 1:10,000 dilution in blocking buffer. Protein bands were visualized following exposure of the membranes to ECL substrate solution (Thermo Fisher Scientific) and quantified by densitometry analysis using Image Studio (LICOR) software.

### Lentiviral transduction.

CaCo-2BBe cells at 70%–90% confluence were transduced with lentiviral particles containing shRNAs targeting STARD7 or a nontarget control shRNA (Mission nontarget shRNA control; MilliporeSigma). STARD7 shRNA and nontarget control shRNA lentivirus were generated by the Cincinnati Children’s Hospital Medical Center Viral Core using a 4-plasmid packaging system. Lentiviral particles were incubated with CaCo-2BBe cells (multiplicity of infection ~10) in the presence of Polybrene (4 μg/mL; MilliporeSigma) for 24 hours followed by selection in puromycin at a concentration (2 μg/mL) that killed uninfected cells within 3 days. The lentivirus-transduced CaCo-2BBe cells (passage 1–5) were grown to confluence under puromycin selection pressure. STARD7 knockdown was assessed by Western blot and immunofluorescence analysis.

### Transmission electron microscopy.

CaCo-2BBe cells were fixed for transmission electron microscopy as described (70). Electron micrographs were collected using an H-7650 TEM (Hitachi High Technologies America, Inc.) equipped with a transmission electron microscope charge-coupled device camera (Advanced Microscopy Techniques).

### Immunofluorescence microscopy.

CaCo-2BBe cells were seeded onto 1.5 glass coverslips in 6-well plates at a density of 600,000 cells per well. The cells were fixed with 4% paraformaldehyde at room temperature for 15 minutes, followed by permeabilization for 5 minutes with a wash buffer (PBS containing 0.1% Triton X-100), then blocked with 10% Normal Goat Serum control (Invitrogen) in the wash buffer for 30 minutes. Coverslips were incubated within staining buffer with primary antibody (1:1,000) for 45 minutes at room temperature. The following primary antibodies were used: α-TOMM20, α-Complex I, and α-STARD7. Coverslips were washed, then incubated in staining buffer with secondary antibody (1:500) for 45 minutes with Alexa Fluor 488 α-mouse antibody and Alexa Fluor 594 α-rabbit antibody at room temperature (115-545-003, Jackson Immuno Research and 111-585-003, Jackson Immuno Research, respectively). Cells were counterstained with Phalloidin (Thermo Fisher Scientific, C34552) and DAPI (Thermo Fisher Scientific, D1306) according to manufacturer protocol. Coverglasses were washed and mounted onto microscope slides using ProLong Diamond Antifade Mountant (Thermo Fisher Scientific, P36970). Cells were imaged at 100× original magnification using a Yokogawa CSU-X1 Spinning Disk confocal microscope (Nikon). Images were analyzed using the open source software CellProfiler (72) and ImageJ (NIH) (73).

### Quantitative PCR.

CaCo-2BBe cells were seeded into 24-well plates. At 50% confluence CaCo-2BBe cells were stimulated with either 40 mM lithium chloride, 10 ng/mL TNF-α, 100 ng/mL IL-6, or 10 ng/mL IL-1β. At 6 and 24 hours following stimulation, the cells were washed with Dulbecco’s PBS, lysed, and collected in 500 μL TRIzol (Invitrogen). RNA was isolated using the Quick-RNA Microprep Kit (Zymogen Research Corporation) according to the manufacturer’s protocol. A total of 1 μg of the purified RNA was reverse-transcribed to cDNA using Superscript II RNase H Reverse Transcriptase (Thermo Fisher Scientific): STARD7 (primer: 5′ AGGAGGAGTTGCAGAGATCTATTA 3′; reverse 5′ GTGTAGGTTCCAAAAACTCGG 3′) and hypoxanthine phosphoribosyl transferase (HPRT) (primer: forward 5′ CAGACTGAAGAGCTATTGTAATG 3′; reverse 5′ CCAGTGTCAATTATATCTTCCAC 3′). mRNA levels were quantified by real-time quantitative PCR with the CFX Duet Real-Time PCR System (Bio-Rad Laboratories) using IQ SYBR Green Supermix (Bio-Rad Laboratories). Results were normalized using HPRT expression of the same cDNA samples and visualized as a relative quantification (2^ΔCT^).

### Ussing chamber.

For ex vivo barrier function experiments, 1 cm of freshly isolated jejunum and colonic tissue was mounted between the hemichambers of the Ussing apparatus. For in vitro barrier function experiments, 500,000 CaCo-2BBe cells were plated on Snapwell or Transwell filters (12 mm diameter, 0.4 μm pore; Corning) and cultured for 10–14 days. TER was measured with an EVOM/Endohm or STX2 electrode (Costar), with correction for filter resistance. For stimulation studies, cells were exposed to 100 μM metformin (Cayman Chemical) basolaterally, and TER was monitored for 24 hours. For permeability studies, Snapwell filters were placed in Ussing chambers, and baseline TER measurements were performed. After addition of 2.2 mg/mL FITC-dextran (4.4 kDa; MilliporeSigma) and 1 mg/mL HRP (40 kDa; MilliporeSigma) to the apical bath, 0.25 mL aliquots were removed from the basolateral bath and replaced with fresh Krebs solution every 30 minutes for 3 hours. HRP concentrations were determined by tetramethylbenzidine detection (BD Pharmingen) and spectrophotometry. Dextran-FITC levels were measured by spectrophotofluorometry (490 nm excitation, 530 nm emission).

### Seahorse assay.

To measure glycolytic and mitochondrial metabolic function in IECs, we used the Seahorse XFe96 Analyzer (Agilent Technologies). We used isolated mitochondria or transduced CaCo-2BBe and plated them in a 96-well Seahorse plate. Wells were washed twice with the appropriate Seahorse assay media (Agilent Technologies) and incubated for 30 minutes in a CO_2_-free incubator at 37°C. Oxygen consumption rate (OCR) was determined using a cell Mito Stress Test kit (Agilent Technologies) according to the manufacturer’s instructions. Oligomycin (2 μM), carbonyl cyanide 4-(trifluoromethoxy) phenylhydrazone (1.5 μM), Rotenone (0.5 μM), and Antimycin (0.5 μM) were used in the assay. Glycolytic function was determined using Glyco stress test kit (Agilent Technologies). Glucose-free media was used in the determination of glycolytic function. Glucose (10 mM), Oligomycin (2 μM), and 2-deoxy d-glucose (50 mM) were used in the glycolysis assay. Cell lysate was collected from 96-well plates after assay, and equal protein content was confirmed using BCA protein assay kit (Pierce, Thermo Fisher Scientific). For chemical inhibitor experiments, 100 μM metformin (Cayman Chemical) was added at the beginning of the experiment. Data were analyzed using Wave 2.6 software.

### In vivo cytokine capture assay.

Systemic TNF-α, IFN-γ, and IL-17a levels were quantified in the serum of mice. Briefly, 10 μg of biotinylated detection antibodies against TNF-α (clone TN3), IFN-γ (clone R4-6A2), and IL-17a (eBio17B7) (all Thermo Fisher Scientific) were injected intravenously into mice, and 24 hours later serum was collected. Luminescent ELISAs were performed using plates coated with a secondary capture antibody as previously described (74).

### Intestinal enteroid and monolayer culture.

Murine small intestinal epithelial colonoids were created and maintained in culture as previously described (75). To generate 2D epithelial intestinal monolayers from murine 3D colonoids, a single-cell suspension was obtained by resuspension in 0.05% Trypsin/0.5 mM EDTA and vigorously pipetting up and down. Trypsin was inactivated by adding 1 mL advanced DMEM/F12–containing 10% FBS. Dissociated cells were passed through a 40 μm cell strainer, then cultured in collagen type IV–coated 48-well tissue culture plates until confluence was achieved (~48 hours). Murine 2D cultures were maintained in LWRN complete media supplemented with 50 ng/mL recombinant human EGF and antibiotics/antimycotic.

### Wound healing assays.

For in vitro experiments, 2D cultures of murine colonoids were subjected to scratch wounding assays as previously described (76). Wound closure was quantified at the indicated time points using ImageJ software (National Institutes of Health), calculated as percentage reduction of cell-free surface area compared with immediately after wounding (*t* = 0).

### In vivo ileal epithelial permeability.

In vivo ileal epithelial permeability at baseline was measured as previously described using an ileal loop model (77, 78). Animals were anesthetized with isoflurane (Fluriso, VETONE) at a constant rate using a rodent anesthesia vaporizer machine (E-Z Anesthesia 7000) and placed on a controlled-temperature heat pad to avoid hypothermia. After disinfection of the abdominal skin, laparotomy was performed by midline incision. A 4 cm length of terminal ileum was exteriorized without rupturing of the blood supply. The loop was gently flushed with warm HBSS plus calcium and magnesium (HBSS plus; Corning Cellgro) to remove fecal contents and facilitate normalization of the volume of contents to allow for comparative analyses between groups. The 4 generated cut ends were closed by ligations using nonabsorbable silk suture 3.0 (Braintree Scientific). The loop was injected with 200 μL (1 mg/mL FITC-labeled dextran [4 kDa] dissolved in HBSS+) using the insertion of a 0.5″, 27-gauge needle. The loop was reinserted in the abdominal cavity; then, the peritoneum and skin were closed. After 2 hours, blood was collected by cardiac puncture prior to euthanasia of the animals by cervical dislocation. FITC-dextran flux was determined by measuring plasma at 488 nm in a microplate spectrophotometer (Epoch Biotek) and Gen5 software.

### RNA-Seq analysis.

RNA-Seq data were obtained from the NCBI GEO database, with accession numbers GSE109142, GSE57945, and GSE117993. Downstream analysis was performed in R, where the read counts were analyzed in IDEP 9.1 and DESeq was used to identify the DEGs (79). DEGs were identified with an adjusted *P* value ≤ 0.05, and at least ≥ ± 1.5-fold reads/kb of transcript/million mapped reads (RPKM), and a heatmap was generated using Python on the normalized scale. Kyoto Encyclopedia of Genes and Genomes pathway analysis was used to identify important pathways altered by DEGs. Statistical analysis was performed using SPSS 17.0. Differential expression was defined with a significant change in expression by limma (80). Heatmaps of gene expression were generated using Morpheus (https://software.broadinstitute.org/morpheus/) and Phantasus (81). GO analysis was performed using Enrichr and gene set enrichment analysis.

### Fecal microbiome analyses.

In brief, DNA was isolated using QIAGEN DNeasy Kit and previously published protocol; the V4 region of the bacterial rRNA gene was amplified and sequenced using previously published protocols (82, 83). For each experiment and sequencing run, a shared community file and a phylotyped (genus-level grouping) file were generated using OTUs binned at 97% identity using the dist.seqs, cluster, make.shared, and classify.otu commands in mothur. OTU numbers were arbitrarily assigned in the binning process and are referred to in association with their most specified level of taxonomy.

### Statistics.

Statistical parameters are defined in the figure legends. Data are presented as mean ± SEM. Data differences were considered significant at *P* < 0.05. Comparisons between 2 groups were made using a 2-tailed *t* test. Comparisons between more than 2 groups were made using 2-way ANOVA and where appropriate were followed with a Tukey’s multiple-comparison test. Correlation analyses were performed using Spearman’s rank correlation coefficient. Statistical analysis was performed in Prism (GraphPad Software).

### Study approval.

All animal studies were approved by the IACUC of the University of Michigan, Ann Arbor, Michigan, USA, and Cincinnati Children’s Hospital Medical Center, University of Cincinnati, Cincinnati, Ohio, USA, and performed in accordance with university guidelines.

### Data availability.

The datasets shown in all the figures are listed in an associated spreadsheet of Supporting Data Values.

## Author contributions

JU, DW, ST, VG, PER, RICS, SM, GI, DNO, LNRT, HLT, AR, MBR, and SE performed experiments. JU, DNO, and ARS performed bioinformatics analyses. JU, TEW, SD, AN, and SPH assisted in study design, analyses, and data interpretation. JU and SPH wrote the manuscript. CAP, MXDO, NWL, YH, LAD, and SD assisted in data analyses, discussion, and drafting the manuscript. SPH supervised and acquired funding. All authors had access to the study data and had reviewed and approved the final manuscript.

## Supplementary Material

Supplemental data

Unedited blot and gel images

Supplemental table 1

Supplemental table 2

Supplemental table 3

Supplemental table 4

Supporting data values

## Version 1 11/22/2024

Electronic publication
