# Supplementary material for: STARD7 maintains intestinal epithelial mitochondria architecture, barrier integrity, and protection from colitis
Source: JCI Insight. 2024 Nov 22;9(22):e172978. doi: 10.1172/jci.insight.172978 (PMC11601949; doi:10.1172/jci.insight.172978)
Supplement: Supplemental data [file jciinsight-9-172978-s019.pdf]

Supplementary Figure 1

A.

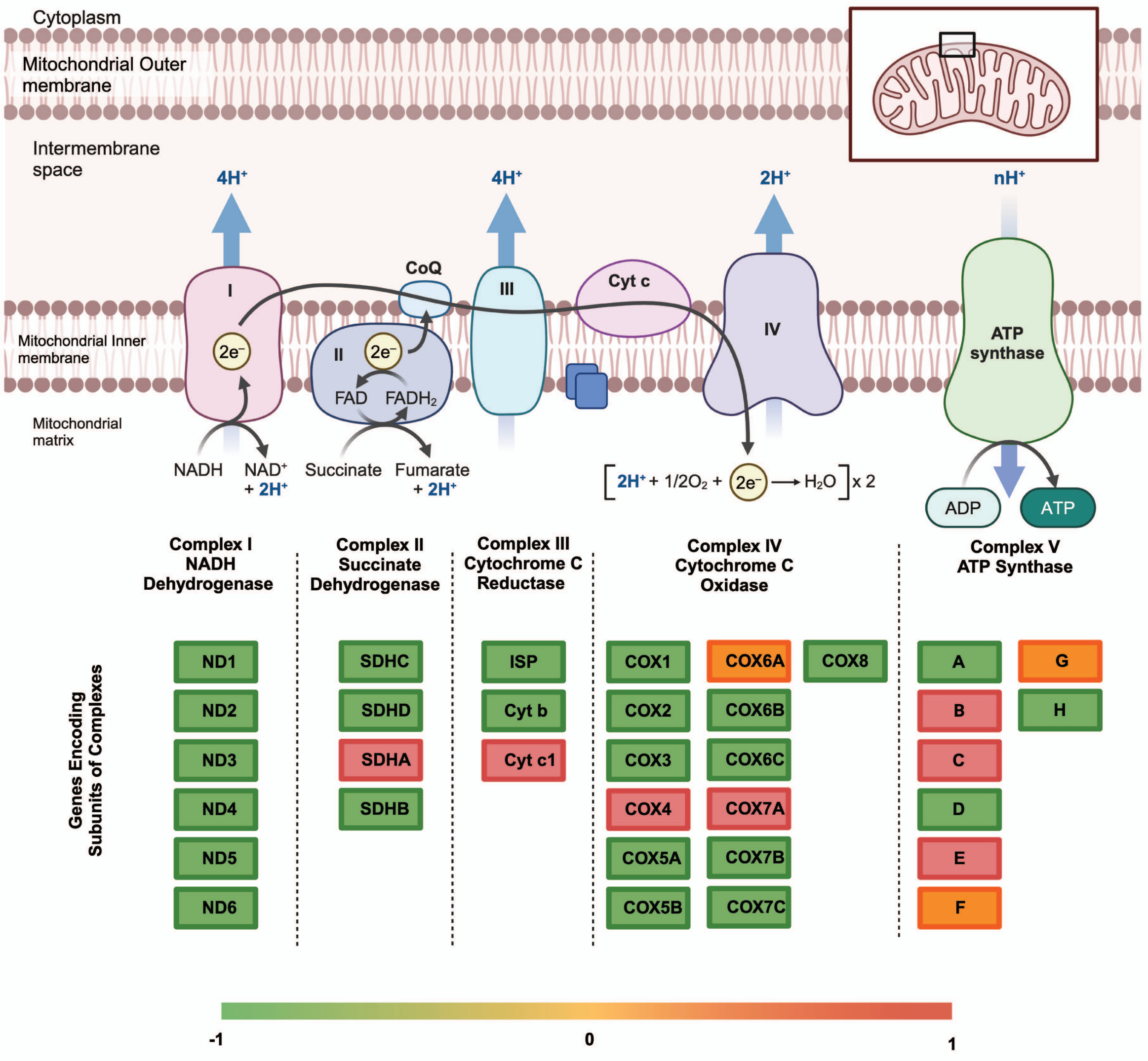

Supplementary Figure S1. DEGs are Enrichment of pathways involved in oxidative phosphorylation in UC. Pathway analysis of differentially expressed genes in Quartile 1 *Stard7* UC patients relative to non-IBD patients involved in oxidative phosphorylation. Stratification of the UC cohort into quartiles based on *STARD7* mRNA RPKM values [Q1 (0-25%), 16.78 – 39.31; Q2 (26-50%), 39.37 – 43.81; Q3 (51-75%), 43.90 – 48.55 and Q4 (76-100%), 48.68 – 64.84] revealed that UC individuals with the lowest *STARD7* mRNA expression [$STARD7^{low}$ (STARD7 Q1)] (GSE109142). Pathway enrichment analyses on the DEGs (up and down; 3238 total number of genes; p adjusted value < 0.05) between $STARD7^{low}$ UC patients (n = 56) from non-IBD patients (n = 16) identified that the downregulated DEGs in the $STARD7^{low}$ UC transcriptome were enriched for expression of genes associated with mitochondrial transmembrane transport, regulation of lipid metabolic process, glucose homeostasis, and transmembrane transport.

Supplementary Figure 2

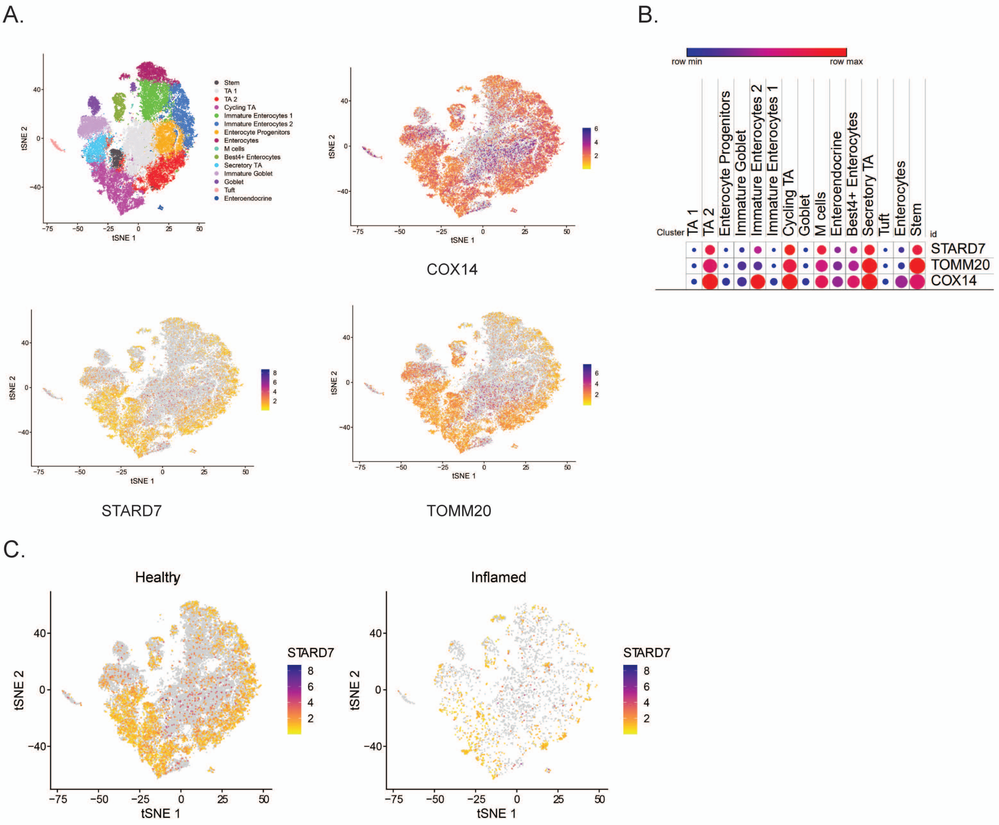

Supplementary Figure 2: Single Cell RNAseq Analysis of IECs Reveals Enrichment for STARD7 in Cell Subsets. (A) tSNE plots of epithelial cells isolated from UC patients (Single Cell Portal accession SCP259). Cell clusters are colored based on cell subsets. Additional heatmaps for expression of STARD7 and mitochondrial genes TOMM20 and COX14 are shown. (B) Dot plots for STARD7, TOMM20, and COX14 across different colonic epithelial subsets. Fractions of expressing cells (dot size) and mean expression level in expressing cells (dot color) are shown. (C) STARD7 expression across epithelial clusters in both inflamed intestine from UC patients and colonic tissue from healthy individuals. (D)

Supplementary Figure 3

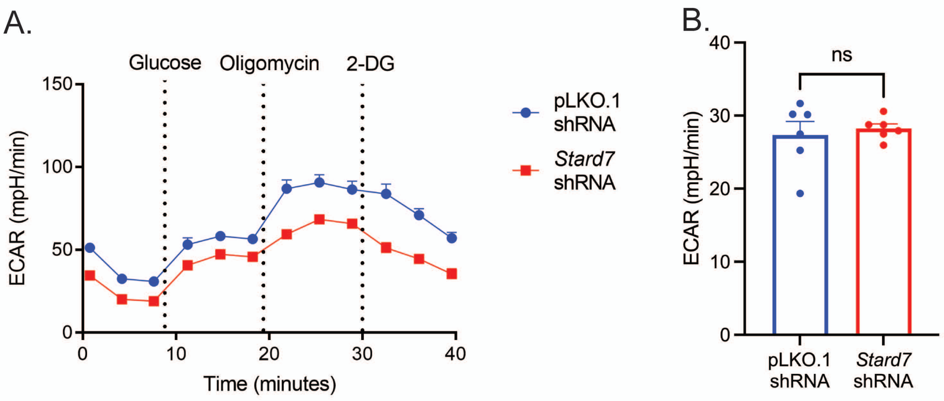

Supplementary Figure 3: STARD7 Deficiency Does Not Impact Glycolysis in Human Colonic Epithelial Cells. (A) Seahorse Glycolysis Stress Test was performed on pLKO.1 shRNA and *Stard7* shRNA transduced Caco-2 BBe cells where extracellular acidification rate (ECAR) was measured over time as cells were exposed at the indicated time points to glucose, oligomycin, and 2-deoxy-D-glucose. (B) Measurement of glycolysis in pLKO.1 shRNA and *Stard7* shRNA transduced Caco-2 BBe cells. Data are representative of at least 2 independent experiments with at least five replicates per group. Data are presented as mean ± SEM. Statistical analysis was performed using unpaired t test.

Supplementary Figure 4

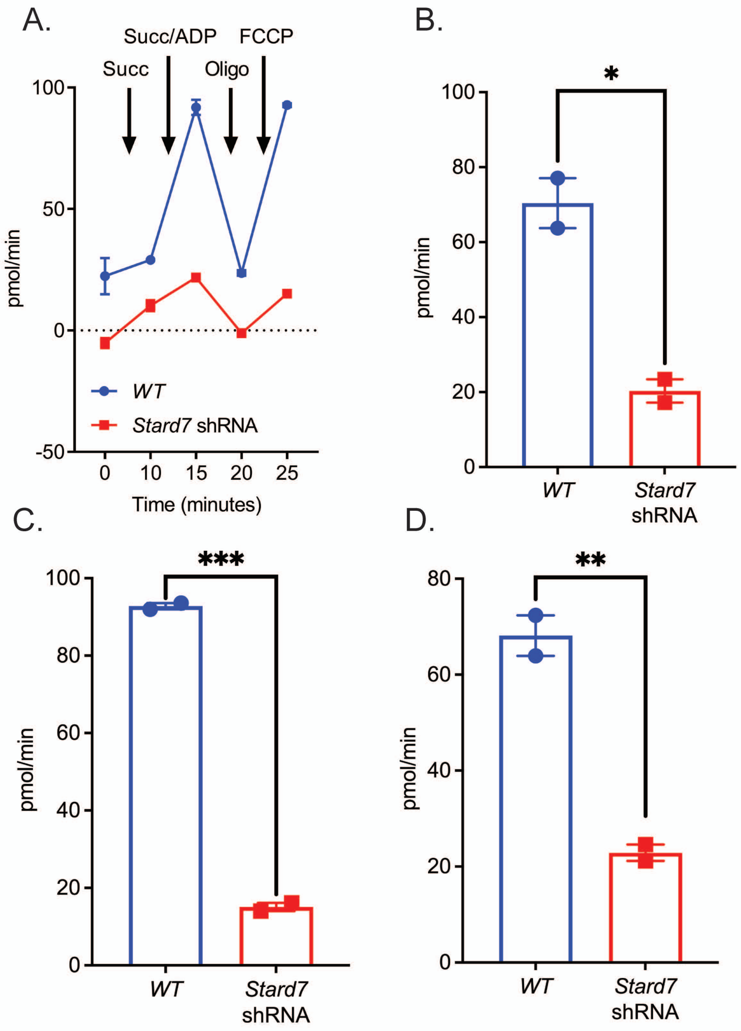

Supplementary Figure 4: Isolated Mitochondria from STARD7 Deficient Human Colonic Epithelial Cells Display Compromised Respiration.
(A) Mitochondria from WT and *Stard7* shRNA transduced Caco-2 BBe cells were isolated measured over time and exposed at the indicated time points to succinate, succinate/adenosine dipshosphate (ADP), oligomycin and carbonyl cyanide p-trifluoromethoxyphenylhydrazone (FCCP) for OCR assessment. Measurement of (B) spare respiratory capacity, (C) maximal respiration, and (D) ATP production in isolated mitochondria from WT and *Stard7* shRNA transduced Caco-2 BBe cells. Data are representative of at least 2 independent experiments with at least two replicates per group. Data are presented as mean ± SEM. Statistical analysis was performed using unpaired t test. *p <0.05, **p <0.01, ***p <0.001.

Supplementary Figure 5

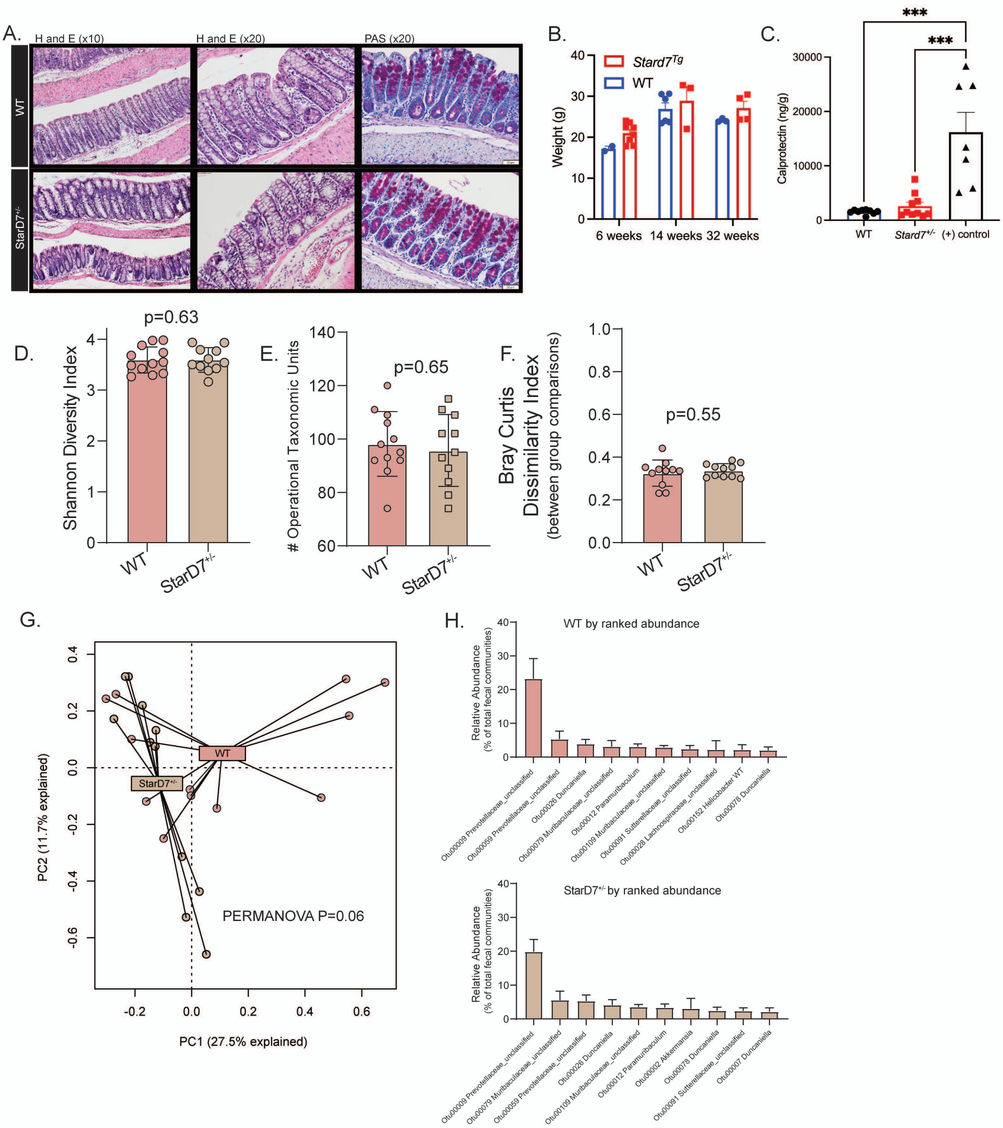

Supplementary Figure 5: AMPK Activation Modulates Susceptibility to Colitis in *Stard7*$^{+/-}$ Mice. (A.) Representative Hematoxylin and Eosin (H and E) and PAS-stained small intestine of age- and strain-matched WT and *Stard7*$^{+/-}$ mice. (B.) Body weight at 6- 14- and 32 weeks of age of age- and strain-matched WT and *Stard7*$^{+/-}$ mice. (C.) Fecal Calprotectin levels in the stool of age- and strain-matched WT and *Stard7*$^{+/-}$ mice. (D.) Measure of alpha diversity in age- and strain-matched WT and *Stard7*$^{+/-}$ mice by Shannon diversity indices, (E.) The number of unique operational taxonomic (OTU) units identified in fecal samples and (F.) Bray-Curtis dissimilarity scores of age- and strain-matched WT and *Stard7*$^{+/-}$ mice. (G.) Principal component analysis (PCA) of 16S data with ordination demonstrating no significant separation of microbial communities between age- and strain-matched WT and Stard7$^{+/-}$ mice. (H.) Shows the most abundant taxa in the fecal pellet of WT and Stard7+/- mice at the OTU level. (B and C) Data are presented as mean ± SEM. Statistical analysis was performed using an unpaired t test. *p <0.05. 16S rRNA amplicon sequencing were used to analyze taxonomic composition and diversity of gut microbiota. (F). A score of 0 represents identical communities and a score of 1 represents no matching or shared taxa. (D – F, n = 11 per group) Permutational analysis of variance (PERMANOVA) where applicable (G), unpaired t test or Mann-Whitney test. (B. and C.) Data are presented as mean ± SEM from n = 2 – 11 mice per group. (D. – F. and H) Data are presented as mean ± SEM. Symbols represent an individual mouse. (D - H). p value indicated.

Supplementary Figure 6

A.

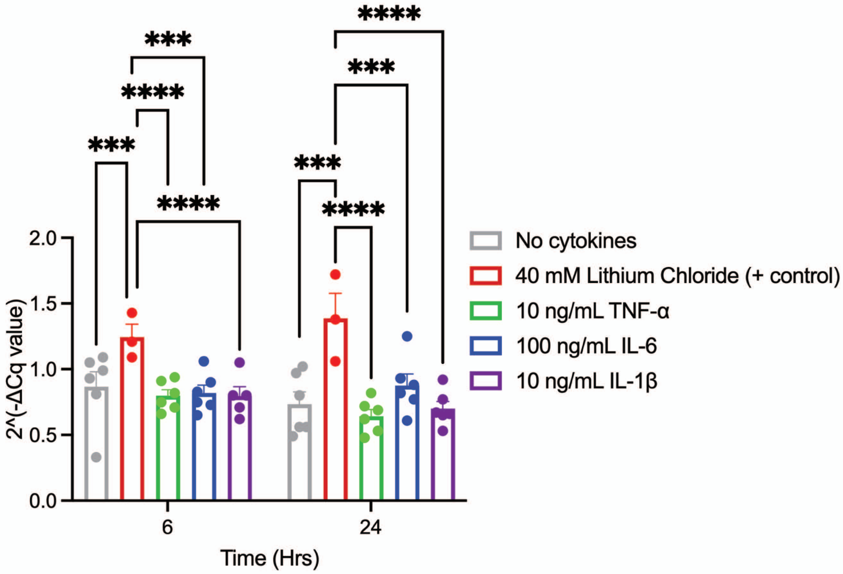

Supplementary Figure 6: The effect of the pro-inflammatory cytokines on STARD7 expression in Human Colonic Epithelial Cells.

STARD7 mRNA expression in TNFα, IL-6 or IL-1β stimulated Caco-2 BBe cells at 6 and 24 hours. Caco-2 BBe cells were stimulated with 10ng/ml TNFα or 100ng/ml IL-6 or 10ng/ml IL-1β. The positive control was Lithium Chloride (LiCl) (40nM). HPRT was used as endogenous control gene for calculation of the delta Cq values. Data are presented as mean ± SEM from n = 6 wells per group. Statistical analysis was performed using unpaired t test. ***p <0.001 and ****p <0.0005.

Supplementary Table 1: RNAseq analyses and Identification of DEGs in UC and CD. (A) Expression values for DEGs between UC and Non IBD individuals. (B) Expression values for DEGs between CD and Non IBD individuals. (C) List of common 74 DEGs between UC and Non IBD individuals and between CD and Non IBD individuals. (D) GO pathway analyses of the common IBD transcriptome Downstream analysis of expressed genes was performed IDEP 9.1.

Supplementary Table 2: UC Patients Stratification by STARD7 Expression. STARD7 quartiles were established based on RPKM values; quartiles Q1 (0-25%), 16.78 – 39.31; Q2 (26-50%), 39.37 – 43.81; Q3 (51-75%), 43.90 – 48.55 and Q4 (76-100%), 48.68 – 64.84. Statistical analysis was performed using SPSS 17.0. The Frequencies procedure was used for the STARD7 quartile analysis.

Supplementary Table 3: Proinflammatory Gene Expression in STARD7^{low} and STARD7^{high} UC Patients. Expression values for innate and adaptive proinflammatory genes in UC patients stratified by STARD7 expression.

Supplementary Table 4: RNAseq analyses and Identification of DEGs in STARD7^{low} UC and Non IBD Individuals. (A) Expression values for DEGs between UC and Non IBD individuals.
