# Supplementary material for: STARD7 maintains intestinal epithelial mitochondria architecture, barrier integrity, and protection from colitis
Source: JCI Insight. 2024 Nov 22;9(22):e172978. doi: 10.1172/jci.insight.172978 (PMC11601949; doi:10.1172/jci.insight.172978)

Western Blot

Unedited gel for Figure 2B

Lane 2 and Lane 6

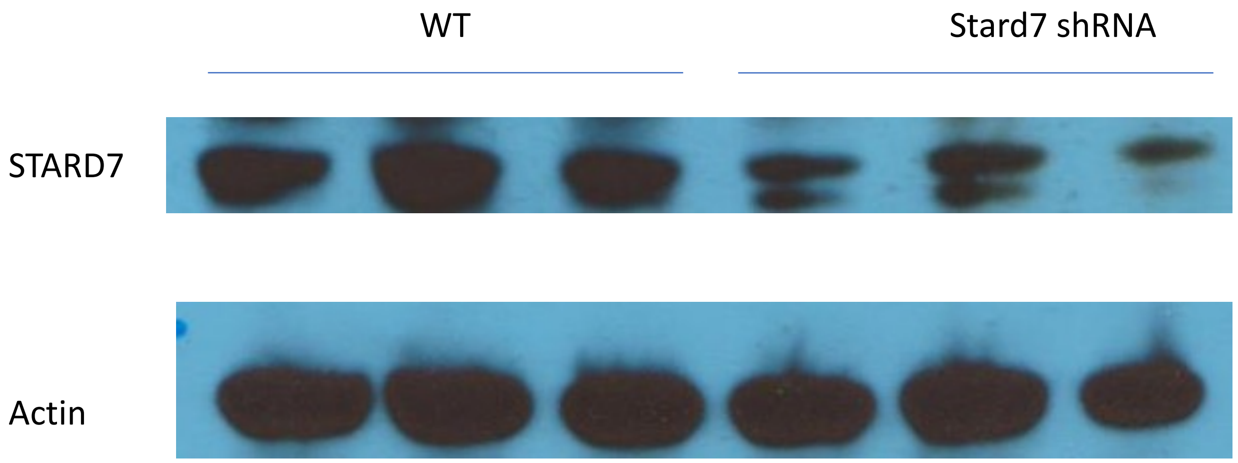

Unedited gel for Figure 2E

Lane 6 and Lane 7

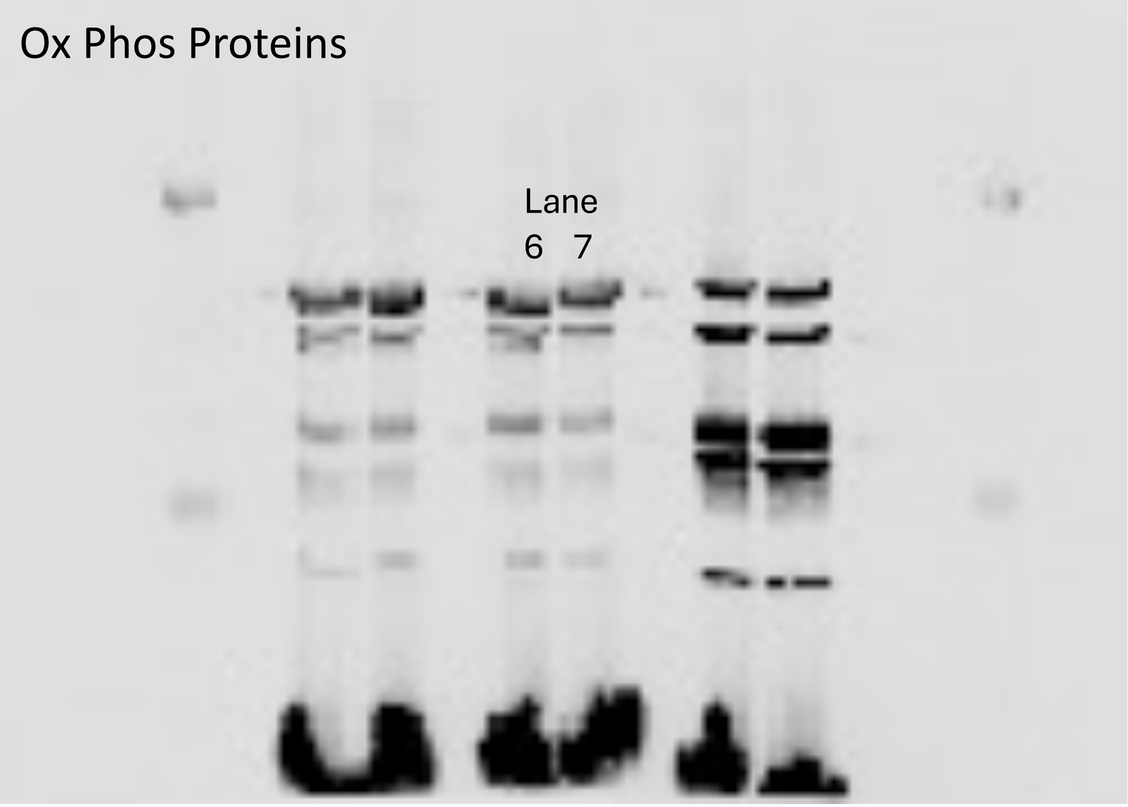

StarD7 and Actin WB

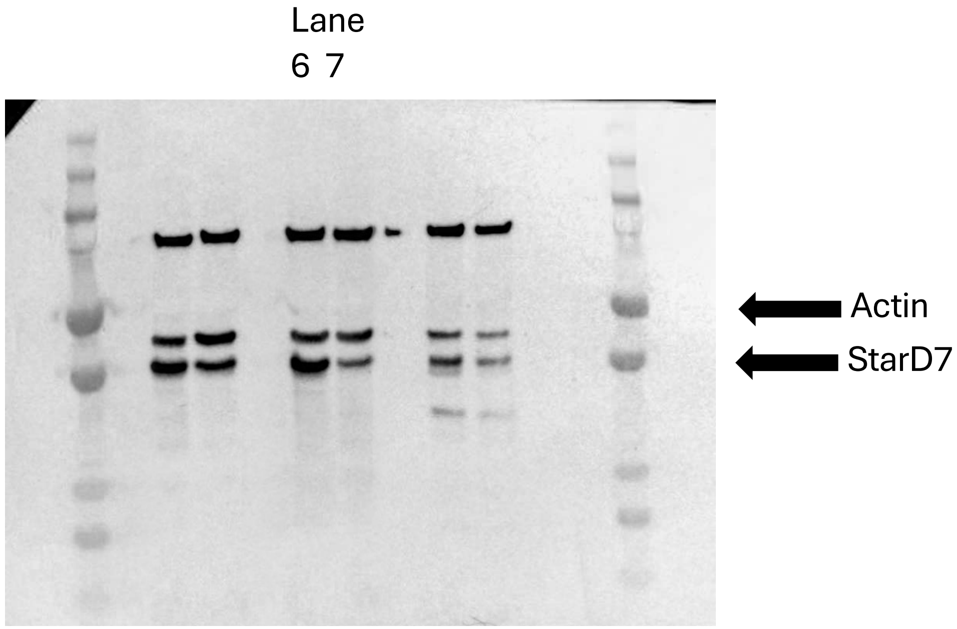

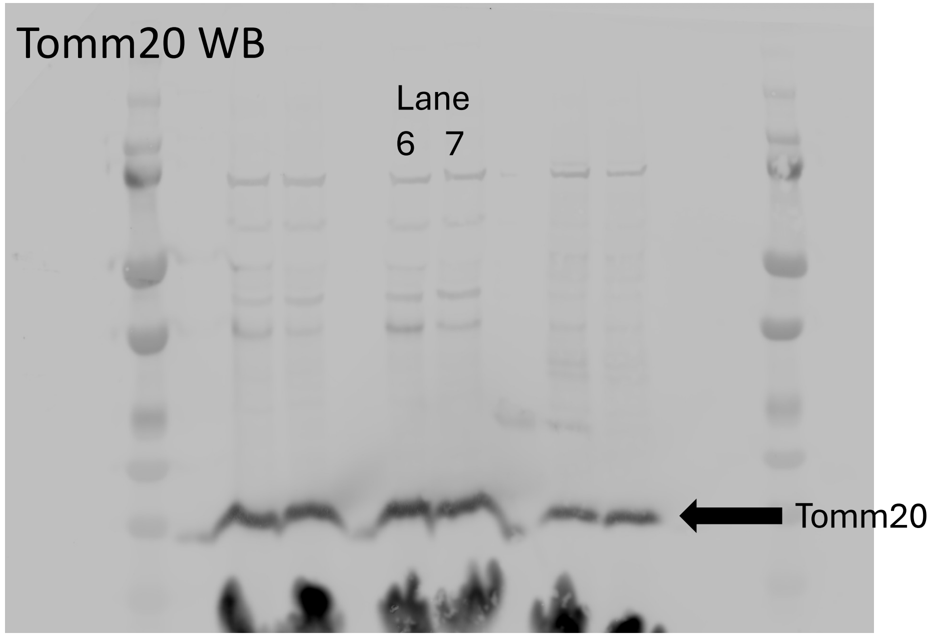

Unedited gel for Figure 3A and B

All lanes used – combined WT and StarD7+/-

WT:
5006
5013
5014

StarD7+/-:
4983
4981
4920

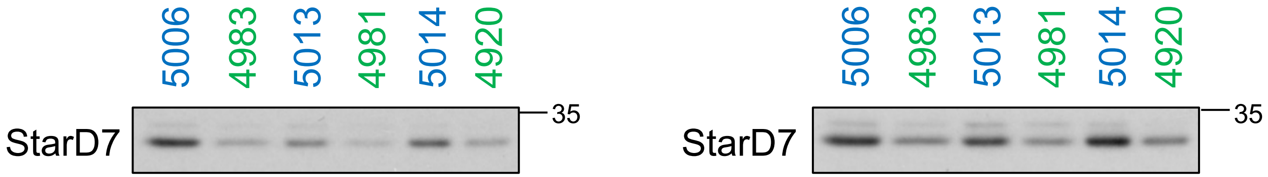

Unedited gel for Figure 3B

WT 5014 and StarD7+/- Lane 4920

WT:
5006
5013
5014

StarD7+/-:
4983
4981
4920

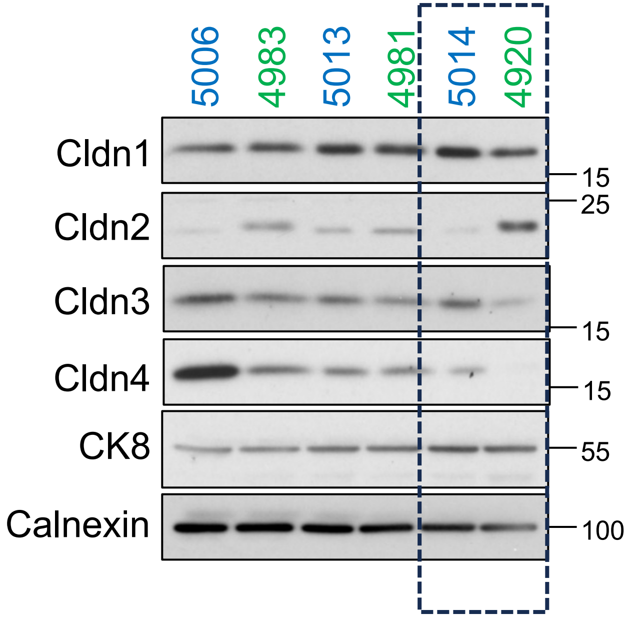

Unedited gel for Figure 3H

Lane 3 and Lane 4

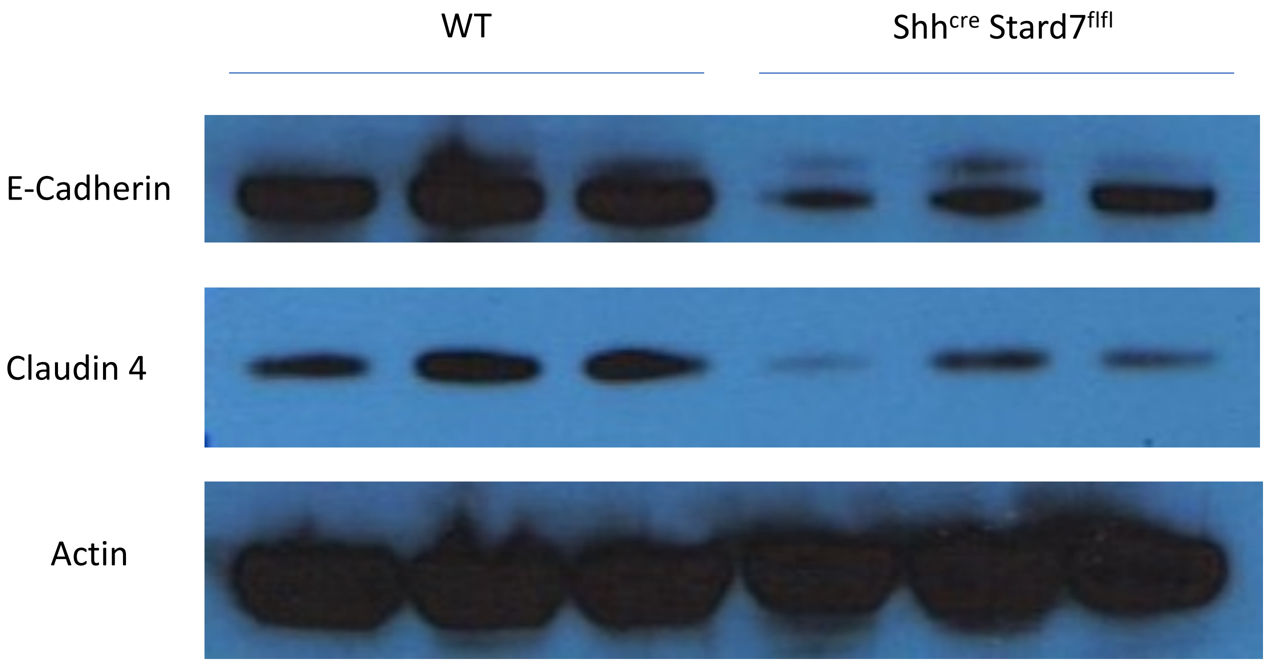

Unedited for Figure 3M

Lane 3 and Lane 4

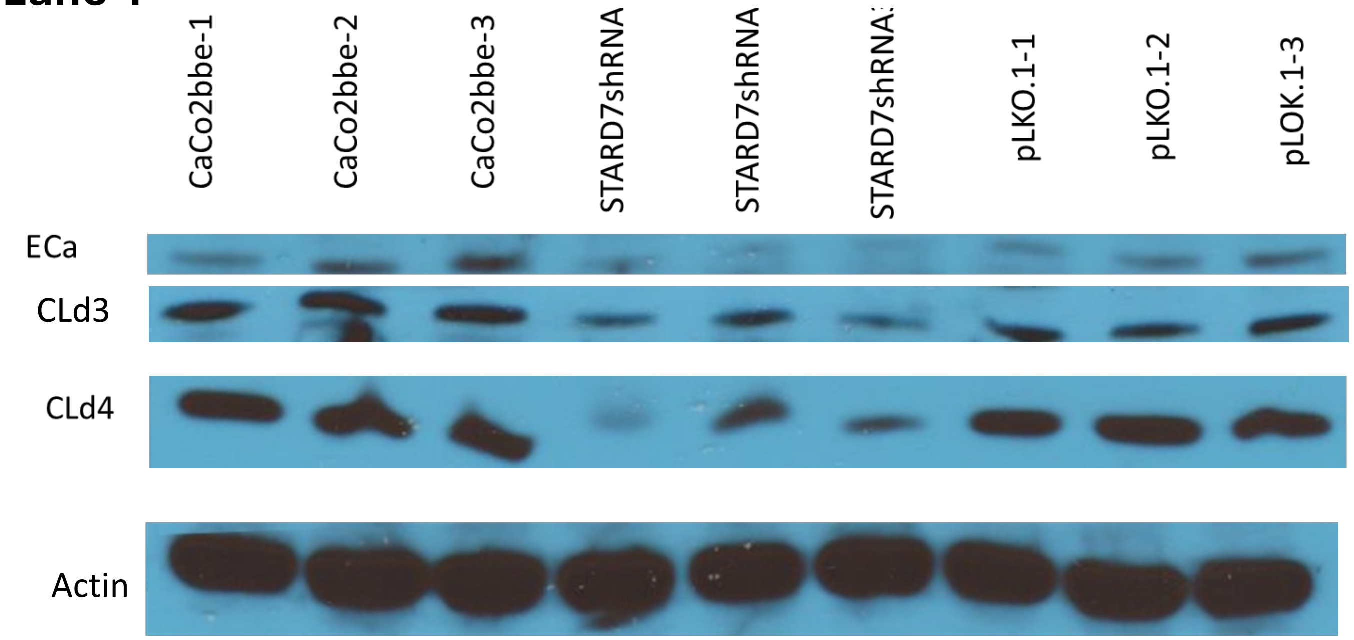

Unedited for Figure 4D

Lane 1 and Lane 5 and Lane 7

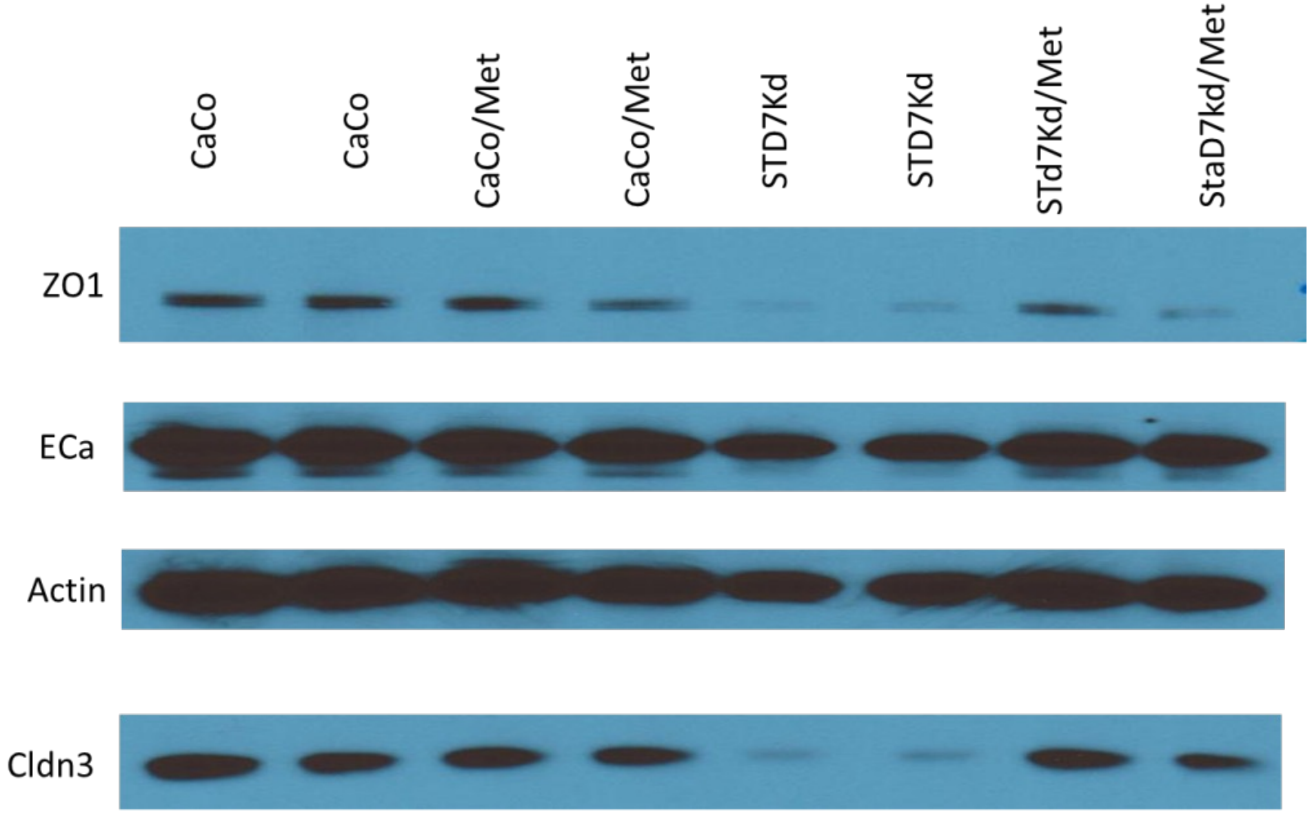

Unedited for Figure 4I

Lane 1-3 and Lane 7-9

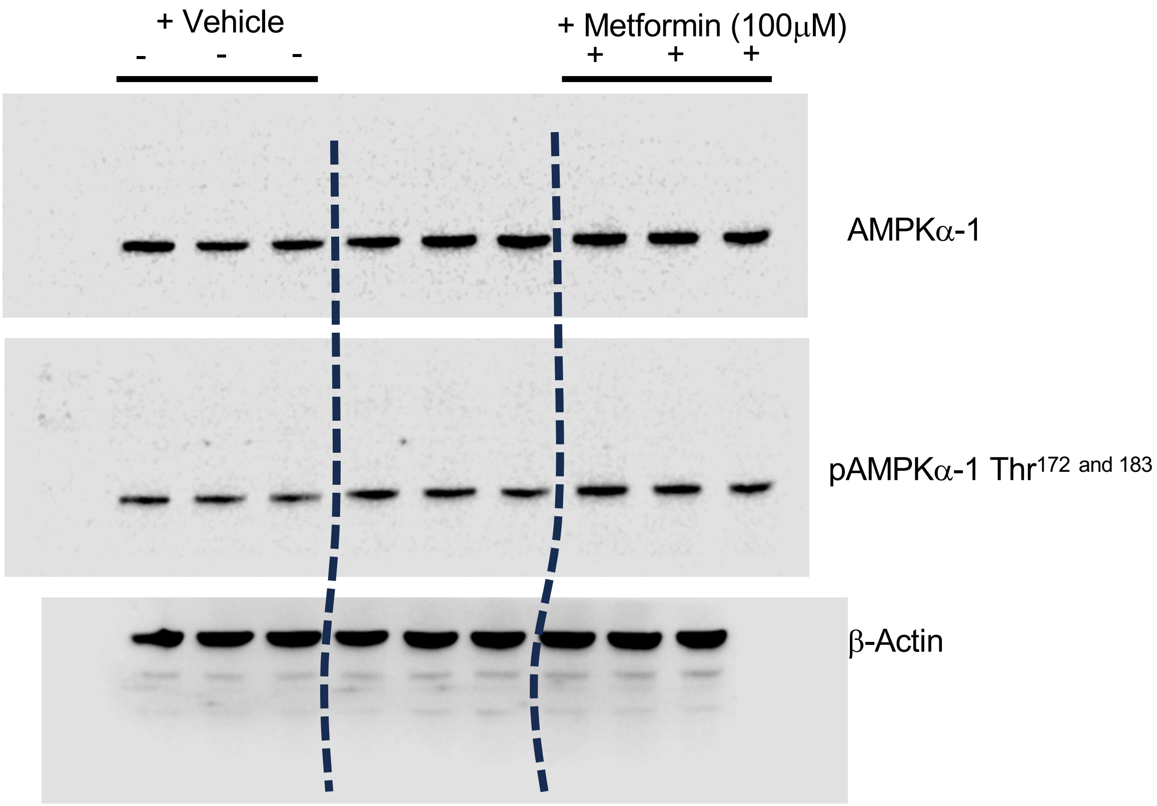

Unedited for Figure 4J

Lane 1-3 and Lane 7-9

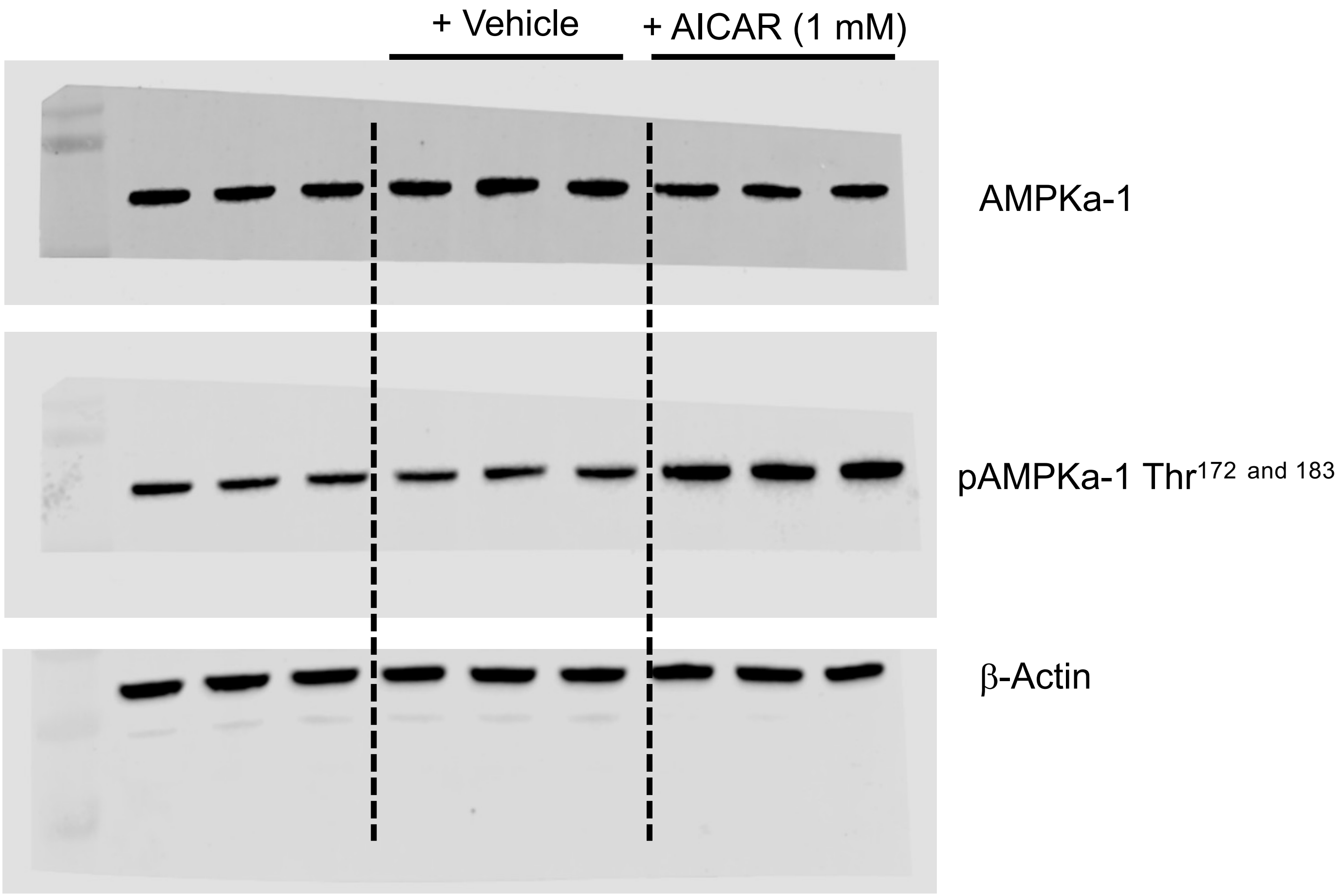

Supplement: Unedited blot and gel images [file jciinsight-9-172978-s020.pdf]
